# Comparative analysis of transcriptome remodeling in plaque-associated and plaque-distant microglia during amyloid-β pathology progression in mice

**DOI:** 10.1186/s12974-022-02581-0

**Published:** 2022-09-24

**Authors:** Anne-Laure Hemonnot-Girard, Cédric Meersseman, Manuela Pastore, Valentin Garcia, Nathalie Linck, Catherine Rey, Amine Chebbi, Freddy Jeanneteau, Stephen D. Ginsberg, Joël Lachuer, Christelle Reynes, François Rassendren, Hélène Hirbec

**Affiliations:** 1 IGF, Univ. Montpellier, CNRS, INSERM, Montpellier, France; 2 Université de Montpellier, CNRS, INSERM, BioCampus UAR3426, Montpellier, France; 3 University Lyon1, CRCL-Centre de Recherche en Cancérologie de Lyon-Inserm U1052-CNRS U5286, Lyon, France; 4 ProfileXpert, SFR santé Lyon-Est, CNRS UMR-S3453, Inserm US7, Lyon, France; 5 LabEx Ion Channel Science and Therapeutics, Lyon, France; 6 Center for Dementia Research, Nathan Kline Institute, Orangeburg, New-York, USA; 7 Department of Psychiatry, Department of Neuroscience & Physiology, and the NYU Neuroscience Institute, New York University Grossman School of Medicine, New York, USA

**Keywords:** Microglia, Alzheimer’s disease, Amyloid plaques, Inflammation, Laser microdissection, RNA-seq, APP^swe^/PS1^dE9^

## Abstract

**Background:**

Research in recent years firmly established that microglial cells play an important role in the pathogenesis of Alzheimer's disease (AD). In parallel, a series of studies showed that, under both homeostatic and pathological conditions, microglia are a heterogeneous cell population. In AD, amyloid-β (Aβ) plaque-associated microglia (PAM) display a clearly distinct phenotype compared to plaque-distant microglia (PCM), suggesting that these two microglia subtypes likely differently contribute to disease progression. So far, molecular characterization of PAM was performed indirectly using single cell RNA sequencing (scRNA-seq) approaches or based on markers that are supposedly up-regulated in this microglia subpopulation.

**Methods:**

In this study based on a well-characterized AD mouse model, we combined cell-specific laser capture microdissection and RNA-seq analysis to *i)* identify, without preconceived notions of the molecular and/or functional changes that would affect these cells, the genes and gene networks that are dysregulated in PAM or PCM at three critical stages of the disease, and *ii)* to investigate the potential contribution of both plaque-associated and plaque-distant microglia.

**Results:**

First, we established that our approach allows selective isolation of microglia, while preserving spatial information and preventing transcriptome changes induced by classical purification approaches. Then, we identified, in PAM and PCM subpopulations, networks of co-deregulated genes and analyzed their potential functional roles in AD. Finally, we investigated the dynamics of microglia transcriptomic remodeling at early, intermediate and late stages of the disease and validated select findings in postmortem human AD brain.

**Conclusions:**

Our comprehensive study provides useful transcriptomic information regarding the respective contribution of PAM and PCM across the Aβ pathology progression. It highlights specific pathways that would require further study to decipher their roles across disease progression. It demonstrates that the proximity of microglia to Aβ-plaques dramatically alters the microglial transcriptome and reveals that these changes can have both positive and negative impacts on the surrounding cells. These opposing effects may be driven by local microglia heterogeneity also demonstrated by this study. Our approach leads to molecularly define the less well studied plaque-distant microglia. We show that plaque-distant microglia are not bystanders of the disease, although the transcriptomic changes are far less striking compared to what is observed in plaque-associated microglia. In particular, our results suggest they may be involved in Aβ oligomer detection and in Aβ-plaque initiation, with increased contribution as the disease progresses.

**Graphical Abstract:**

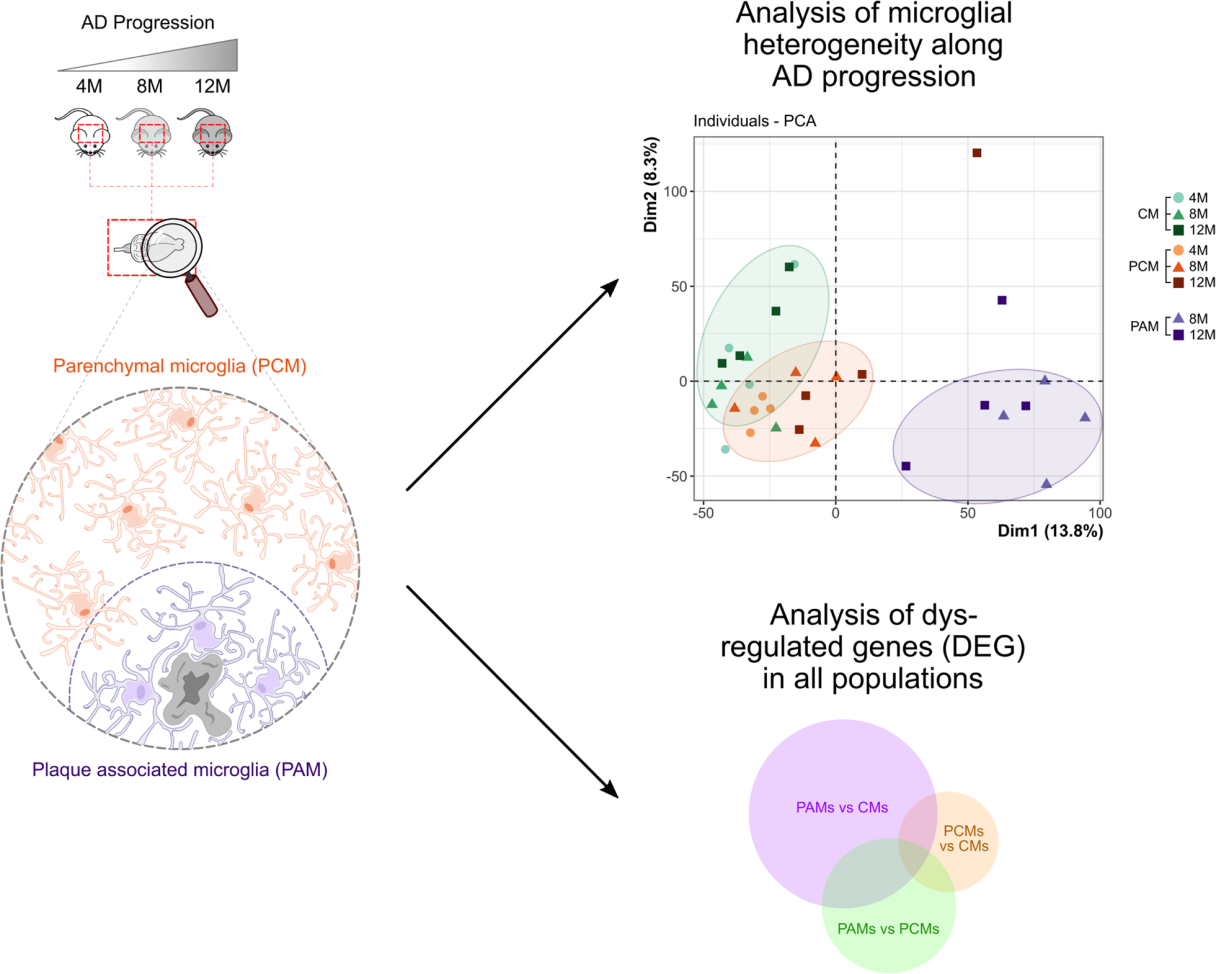

**Supplementary Information:**

The online version contains supplementary material available at 10.1186/s12974-022-02581-0.

## Background

Alzheimer’s disease (AD) is a progressive neurodegenerative disorder and the most common cause of dementia. It affects around 50 million people worldwide and incidence is expected to double within the next 20 years. From a histological point of view, the disease is characterized by several pathological hallmarks, including amyloid-β (Aβ) plaques, neurofibrillary tangles composed of tau, loss of synapses, dystrophic neurites and neuroinflammation [1]. The chain of events leading to AD has been the focus of intense research for decades, but remains poorly understood. Based on the sequential appearance of the hallmarks, it has been proposed that Aβ deposition represents an initiating event in the cascade that ultimately leads to neuronal degeneration [2]. However, recent results suggested that Aβ accumulation alone is insufficient to explain the disease progression [3].

Recent human genome wide association studies (GWAS) revealed that many risk factors for AD are associated with genes that are either highly, preferentially or exclusively expressed by microglia [4–7]. These data, together with the identification of profound remodeling of the microglia transcriptome in AD mouse models [5–7], support the hypothesis that microglia are key contributors to AD pathogenesis. They also open the therapeutic potential of modulating microglial functions for AD treatment. However, the role of microglia in AD is still unclear and heavily debated, with conflicting reports regarding their protective or detrimental impact.

Microglia are the innate immune cells of the brain where they have key homeostatic functions. In the adult brain, they notably provide trophic support for neurons, regulate neuronal excitability, and actively monitor the brain parenchyma [8]. Yet, when brain homeostasis is disrupted microglia respond. Once believed to be an all-or-nothing process, it is now well established that microglia reaction is a complex and dynamic biological process which depends on many factors including brain region vulnerability, sex, and nature of the trigger and the duration of the triggering event. Further, high-content molecular studies have established that both homeostatic and reactive microglia are indeed very diverse [9, 10]. Single-cell RNA-seq (scRNA-seq) studies have been instrumental in deciphering the molecular diversity of microglia in neurodegenerative disease. In AD, these approaches have allowed the identification of several microglia subtypes linked to the disease [11–13]. The disease-associated microglia (DAM; [11]), the late response microglia (LRM; [12]) and the activated-response microglia (ARM; [13]) appear closely related. Others microglia subtypes have also been described in AD including, the early response microglia (ERM; [12]), the interferon-response microglia (IRM, [13]), and the transiting response microglia (TRM, [13]), but their roles remain elusive. Further, using single-nuclei RNA-seq (snRNA-seq) approach, Gerrits et al. [14] revealed microglia diversity in the brain of AD patients and identified two AD-associated microglia subtypes, AD1 and AD2, which associate with Aβ and Tau pathology, respectively.

In addition to their molecular diversity, microglia cells in AD display a clear phenotypic heterogeneity. Aβ-plaque-associated microglia, here referred to as PAM, exhibit strong morphological alterations. More specifically, they present a reduced average surface area and branching that is a morphological characteristic typical of reactive microglia [15]. PAM also undergo important channel activity changes, and display altered phagocytic properties [15, 16]. In contrast, plaque-distant microglia, here referred to as PCM, display only minor morphological alteration compared to control microglia [15]. Still, in the late stage of the disease, PCM display an intermediate inflammatory profile compared to either non-AD control, here referred to as control microglia (CM), or plaque-associated microglia [17]. However, in general, plaque-distant microglia have been less well studied in AD; and whether the observed subtle morphological changes are associated with functional changes in this subtype has not been clearly defined. Adding to the complexity, significant temporal diversity in microglial morphology has been observed, in both mouse models and patients, during AD progression [18].

The molecular signature of PAM has only been indirectly inferred. First, based on the expression of a handful of markers, molecularly defined DAM, ARM and LRM have been proposed to correspond to the spatially defined PAM [11–13]. Second, based on initial assumptions that PAM are Class II major histocompatibility complex positive (MHCII+) [17] or are actively Aβ-phagocytosing cells [19], more recent studies used fluorescent-activated cell sorting (FACS) to characterize the transcriptome of PAM compared to PCM (i.e., MHCII- or Aβ-non-phagocytosing microglia). However, these latter approaches required tissue dissociation prior to cell sorting, a process that interferes with the assessment of the disease-driven microglia transcriptomic changes [20]. Finally, other studies used laser capture microdissection approaches to analyze the transcriptomic changes in plaque-associated and/or plaque-distant tissues, thus capturing transcriptomic changes arising in heterogeneous cell types [6, 21, 22]. However, the transcriptomes of PAM and PCM have never been directly established.

To overcome these limitations, we combined cell-specific laser capture and RNA-seq analysis to investigate the functional roles of both PAM and PCM. First, we established that this approach is well suited to study the transcriptome remodeling of spatially distinct microglia. We then identified, in both microglia subpopulations, networks of co-deregulated genes and analyzed their potential functional roles in AD. Moreover, by investigating the microglia transcriptomic remodeling at early, intermediate and late stages of the disease, we were able to highlight the dynamics of these processes. Our comprehensive study confirms that proximity to Aβ-plaques dramatically alters the microglia transcriptome and reveals that these changes can have both positive and negative impacts on the surrounding cellular network. Our approach also allowed us to study plaque-distant microglia and to reveal that PCM are not bystanders to the disease progression and may be involved in Aβ oligomer detection and plaque initiation, with their contribution increasing as the disease progresses.

## Methods

### Animals

The APP^swe^/PSEN1^dE9^: CX3CR1^+/eGFP^ mice, referred to as APP/PS1-CX3, used in this study were obtained by crossing heterozygous APP^swe^/PSEN1^dE9^ male mice with female CX_3_CR_1_^eGFP/eGFP^. APP^swe^/PSEN1^dE9^ mice were purchased from the Jackson Laboratories [23, 24] and subsequently bred under the C57BL6/J background in the Specific Pathogen Free animal facility of the Institute for Functional Genomic (IGF, Montpellier, France; Agreement from the Ministry of Agriculture N° D34-172-13). CX_3_CR_1_^eGFP/eGFP^ mice were generous gift from Dan Littman [25] and were also maintained in the IGF facility under the C57BL6/J background. Comprehensive characterization of the APP^swe^/PSEN1^dE9^: CX3CR1^+/eGFP^ has been performed previously [26] and revealed that Cx3cr1 haplodeficiency has little impact on the disease progression and that APP/PS1-CX3 mice are a useful model to study microglia in AD-like pathology. Mice were housed in a 12-h light–dark schedule with food and water available ad libitum. All experiments followed European Union (Council directive 2010/63/UE) and institutional guidelines for the care and use of laboratory animals. The animal experiment protocols used in this study were approved by the Comité d'Ethique pour l'Expérimentation Animale Languedoc Roussillon (CEEA-LR; APAFiS#5252). Experiments were performed in 4, 8- and 12-month-old (mo) animals (see Additional file 14: Table S2 for details on the mice used).

### Human brain samples

The human brain samples used in the present study were a generous gift from Dr Stephen D. Ginsberg. Brain tissues accession and clinical pathological assessment are further described in detail in [27–29]. In the present study, we used unfixed tissue stored at − 80 °C. Unfixed frozen tissues of the prefrontal cortex [Brodmann areas 9–10] from (n = 33) subjects stored at -80 °C were employed for qPCR as described previously [27–29]. A total of (n = 19) control subjects with no cognitive impairment (NCI) (11 M/8 F) and (n = 14) AD cases (6 M/8 F) were used. Neuropathology was determined by a board certified neuropathologist blinded to the clinical diagnosis. A summary of case materials is summarized in Additional file 23; Table S11.

### Tissue sample preparation

All solutions mentioned hereon were prepared using RNAse-free buffers.

#### Laser capture microdissection

The protocol used for tissue preservation was validated after a series of experiments the results of which are summarized in Additional file 13: Table S1. Four evaluation criteria were selected for protocol validation: *i*) GFP + cells visualization, *ii*) quality of the cryostat slices obtained; *iii*) RNA integrity and *iv*) RNA yields. The approach selected offers the best compromise between all four criteria. After induction of deep anesthesia with 2 µg/g pentobarbital solution (Euthasol Vet, TVM), mice were perfused intracardially with 20 ml of phosphate buffer saline (PBS; Ambion), followed by 20 ml PBS solution containing 20% sucrose (Sigma-Aldrich, S7903). The brain was then removed, immersed overnight in PBS solution containing 30% sucrose and then flash frozen in − 40 °C Isopentane (Merck, #320404). Brains were stored at − 80 °C for at least 24 h and for up to 6 months. 8-μm-thick coronal sections were cut using a cryostat (Leica) with chuck and cabin temperatures were maintained at − 25 °C. Sections were mounted onto Superfrost slides and then stored at − 80 °C for up to 2 days before laser microdissection.

RNAscope: After induction of deep anesthesia with 2 µg/g pentobarbital solution (Euthasol Vet, TVM) mice were transcardially perfused with 10 ml cold PBS. Brains were extracted, fixed in 4% paraformaldehyde (PFA, Sigma, P6148) for 2 h at room temperature (RT) and post-fixed overnight at 4 °C in fresh 4% PFA. Tissues were then cryoprotected by successive immersion in PBS solutions containing increasing sucrose (Sigma, S7903) concentrations (i.e., 10%, 20% and 30%). Tissues were transferred from one solution to the next when the brain sank indicating equilibrium between the tissue block and the solution. Brains were included in OCT (TissueTek, #4583), flash frozen in − 50 °C Isopentane (Merck, #320404), and stored at − 80 °C for at least 24 h. 14 μm thick serial coronal sections were cut using a cryostat (Leica), directly mount onto Superfrost slides and stored at − 80 °C until use. For RNAscope on postmortem human prefrontal cortex, 20 μm thick serial sections were cut from frozen BA9-10 tissue blocks using a cryostat (Microm, NX70), and directly mounted onto Superfrost slides and stored at − 80 °C until use.

### Laser capture microdissection

#### Thiazine red staining for Aβ plaque detection

Thiazine red (TR) is an analog of naphthol-based azo structures which binds β-pleated sheet structures. Like Thioflavin-S, it stains dense-core plaques but with maximum emission at 580 nm [30]. On the day of microdissection, slides were removed from freezer, immediately placed for 1 min in 70% ethanol solution and then stained by immersion for 1 min in 75% Ethanol solution containing 0.165% TR. Excess TR was removed by performing three 15 s with 75% Ethanol solutions. Dehydration was continued by successive immersion for 1 min in ethanol solutions of increasing concentration (VWR, #20281.310; 95%, 100% and 100%), followed by two immersions of 5 min each in 100% xylene (VWR, #289751.291). Then, slides were allowed to dry in a vacuum bell for at least 1 h. Hygrometry in the microdissection room was controlled throughout the procedure and, to ensure preserving the sample quality, slides were used within 3 h after removal from the vacuum bell. Microglial cells (identified as GFP expressing cells) and TR staining were visualized at 20× magnification using the Nikon Eclipse Ti-E epifluorescence microscope which equipped the PixCell IIe Laser Capture Microdissection system (Applied Biosystems/Excilone Elancourt, France). GFP+ cells of the cerebral cortex were laser captured in CapSure HS LCM Caps (Arcturus/Life Technologies). Laser characteristics were set at the smallest “spot” size (i.e., 7.5 µm); the power of the Infra-red laser, the number and the durations of the pulses were adjusted for each slide. At the end of the session the captured cells were immediately lysed in the RLT-plus buffer (Qiagen; #1053393) and stored at − 80 °C. In AD-CX3 mice: plaque-associated microglia and plaque-distant microglia were isolated from the same mice with TR staining used to discriminate both microglia subtypes. Thus, PAM corresponded to microglia located within 70 µm of the center of a dense-core Aβ plaque whereas PCM were microglia located further than 100 µm from the center of any dense-core Aβ plaque (Additional file 1: Fig. S1). Distances were measured in 2D sections and chosen based on the average size of the plaques (i.e., 12–20 µm in diameter) and on preliminary observations showing that microglia clustered around plaques were located within 70–80 µm of the center of the plaque (not shown). Additionally, when selecting PCM, we excluded microglia that, even in the absence of TR staining, appeared clustered. An extended description of the laser capture microdissection procedure is provided in the “Extended methods” section of the manuscript.

### RNA extraction and RNA-seq

LCM-isolated microglia from at least 4 microdissected sections in at least 2 independent sessions were pooled. This totaled about 400 microglial cells for each mouse and experimental condition. We used 4 mice per condition, n=32 mice in total (for details see Additional file 14: Table S2). RNAs were extracted using the Qiagen RNeasy Plus micro kit (Qiagen, #74034) following a protocol slightly adapted from that of the manufacturer. RNAs were eluted in 16 µl RNAse-DNAse free H_2_O. Total RNA quality was verified by extracting RNA from the tissue remaining on the slide after microdissection and determining its integrity using the Agilent 2100 Bioanalyzer (Agilent). All RNAs had RNA Integrity Numbers (RINs) higher than 8.0.

Library preparation and RNA sequencing were performed by the ProfileXpert core facility (Lyon, France). In brief, mRNAs were pre-amplified from 200 pg total RNA using the SMART-Seq V4 Ultra Low RNA kit (Clonetec). Library preparation was performed from 500 pg cDNAs using the Nextera kit (Illumina) following manufacturer instructions. Libraries were sequenced using an Illumina NextSeq500 platform and 75 bp single-end sequencing data were obtained with between 28 and 41 million reads per sample. Perfect trimmed reads were aligned to Mus musculus mm10 reference genome using the TopHat2 software [31]. The featureCounts tool was used to determine the number of reads mapping to each gene [32].

### qPCR on human postmortem brain samples

RNA from human postmortem AD and control brain samples were extracted using the Qiagen RNeasy Plus Mini kit (Qiagen, #74136) following manufacturer protocol. Quality of the total RNA was assessed using the Agilent 2100 Bioanalyzer (Agilent). All samples had RNA Integrity Numbers (RINs) higher than 6.4. Reverse transcription of 500 ng total RNA was performed using the iScript kit (BioRad). Real-time PCR was performed in 384-well plates in a final volume of 10 µl using SYBR Green dye detection on the LightCycler480 system (Roche-Diagnostic). The primers pairs for CLEC7A, CST7 and CYBB were designed using Primer 3 software.  These sequences are reported in the Extended Method section. All three analyzed genes showed detectable expression levels. Because *i*) these three genes are specifically expressed in microglia and *ii*) the objective was to assess their potential up-regulation in microglia, HEXB was selected as normalizing gene. The results were expressed as Cq, normalized to the Cq value of the HEXB (-ΔCq), with final results are presented as the percentage of the mean of the controls as described previously [27].

### Bioinformatics analyses and networks

Bioinformatics and statistical analyses were performed in collaboration with the StatABio facility (BioCampus UAR 3426 CNRS-US 09 INSERM-UM) using R software (3.6.0).

#### Differential gene expression

Samples to be included in the different analyses were selected according to the question addressed. After selection, gene expression normalization was performed using Relative Log Expression (RLE) normalization implemented in the DESeq_2_ R package and genes with less than 1 count per million (cpm) in at least 3 out of 4 replicates and at least one condition, were filtered out. To detect the differentially expressed genes (DEG), we applied Generalized Linear Models (GLM) with tagwise dispersion. Both raw- (pv_raw_) and adjusted-P values (pv_adj_) were computed.

Principal component analysis (PCA) was performed using FactoMiner R package [33].

#### Weighted gene co-expression network analysis (WGCNA)

WGCNA R software package was applied to identify co-expression modules among pre-selected genes [34]. In brief, mean connectivity and scale dependency measures were calculated to choose the proper soft power and to reconstruct the network. Soft threshold power was then evaluated using network analysis functions to preserve more correlated genes based on scale-free topology [35]. Identification of the potential modules was performed by applying the module analysis algorithm to the dissimilarity matrix. The minimal number of genes in each module was set to limit the number of unassigned genes. In practice, unassigned genes represented 3–11% of the genes’ selection. The extracted modules were labeled with colors, with turquoise being the most abundant, blue the second most abundant and brown the third most abundant modules. Unassigned genes were placed in the grey module. Eigenvalues, which can be seen as the expression values of “artificial genes” quantifying the expression variations of the genes within a particular module, were also calculated.

#### Gene network representations

Gene networks of pre-selected genes were constructed using specific applications (Apps) implemented in the Cytoscape software. First, we used the *STRING* App, with full STRING network and 0.7 confidence cutoff as settings to construct the network. At this stage, isolated genes were removed. Large networks were further divided into subnetworks using the *MCL* tool of the *STRING* App. To identify hub genes, we then ran the *CytoHubba* app, employing five calculation methods: Degree, Edge Percolated Component (EPC), EcCentricity, Maximal Clique Centrality (MCC), and Maximum Neighborhood Component (MNC). The intersecting genes derived using these five algorithms encode the most highly connected proteins and may represent key candidate genes with important biological regulatory functions. Finally, we used the *Omics Visualizer* app to display gene expression values changes on the generated networks.

#### Microglia gene signature enrichment

To assess the extent of which list of pre-selected genes overlapped with mouse and/or human microglia gene signatures that were previously defined, we performed overrepresentation analysis (Sensome [36]; DAM [11]; microglial neurodegenerative phenotype (MGnD) [5]; Reactome & New-Sensome, [37]; IAM [38]; ARM & IRM [13]; PIGs [21]; AD1 & AD2 [14]). With these analyses, we determined both the enrichment factor and the associated p-value using the Fisher exact test.

#### Functional enrichment analysis

To study the biological mechanisms and gene ontology of the selected genes, we used the g:Profiler software (https://biit.cs.ut.ee/gprofiler/gost). The Gene Ontology (GO)-biological processes associated with the selected genes were listed; nodes (GO-biological process) with adjusted p-value less than 5% were reported as important. To avoid overly specific and general processes, only GO-biological processes with a size between 30 and 300 were considered. When the large number of GO-terms are affected, redundancy between them is high making it difficult to read graphs. To visualize and interpret those results, we used the *EnrichmentMap* and *AutoAnnotate* apps in Cytoscape to visualize the GO-terms network [39]. When too few biological processes were detected, larger GO-biological processes (up to 500 genes) were also considered. To get further functional insights, Kyoto Encyclopedia Gene and Genomes (KEGG [40]) and Reactome [41] databases were also used to perform pathway enrichment analyses.

### RNAscope (smFISH)

Detection of mouse *Cst7*, *Clec7a* and *Cybb* and human *CLEC7A* and *P2RY12* transcripts was performed on fixed, frozen sections (see Tissues preparation section) using Advanced Cell Diagnostics RNAscope® Multiplex Fluorescent V2 kit. The RNAscope® Probe probes were, respectively, Mm-Cst7 probe (ACD, Cat No. 498711), Mm-Clec7a (ACD, Cat No. 58264), Mm-Cybb (ACD, Cat No. 403381), hu-CLEC7A (ACD, Cat No. 511761-C3) and hu-P2RY12 (ACD, Cat No. 450391-C2). Negative controls were performed in parallel on serial sections using RNAscope® 3-plex Negative Control Probe (ACD, Cat No. 320871).

Hybridization protocol was adapted from that ofthe manufacturer ACD. For mouse brain sections, slides were thawed at RT for 10 min, washed with PBS for 5 min before dehydration in 50%, 70% and 100% Ethanol, 5 min each. For human brain sections, after removal from − 80 °C, slides were rapidly rinsed in PBS, fixed for 1 h in PFA 4% at RT, and then washed with PBS for 5 min. Sections were then dehydrated in ethanol (as above), air-dried for 5 min and exposed to UV light for 10 min to help quench autofluorescence associated with  lipofuscins. Following these preparatory steps, mouse or human slides were baked at 37 °C for 1 h. H_2_O_2_ treatment, target retrieval, protease treatment, probe hybridization and signal amplification were performed according to manufacturer’s instructions. Opal 570 (Perkin Elmer; 1/500 in TSA buffer) was used to detect Clec7a, Cybb, Cst7 and CLEC7A RNAscope® probes, and Opal 520 (Perkin Elmer; 1/500 in TSA buffer) to detect P2RY12 RNAscope® probes. smFISH on mouse sections was followed by histological staining with Thiazine red to detect amyloid-β plaques and immunohistochemistry with GFP to label microglia. smFISH on human sections was followed by immuno-histological staining with the 6E10 antibody to detect amyloid-β pathology. In brief, following incubation for 1 h in PBS containing 10% donkey normal serum (Gibco, S30-100 ml) and 0.3% Triton X-100 to permeabilize and block unspecific labeling, sections were incubated for 48 h at 4 °C in a humidified chamber with anti-GFP (1:2000; Sigma, SAB4301138) or 6E10 primary antibodies (1:500, #803,001, BioLegend). Sections were then washed 3 times in PBS and incubated for 2 h at RT with the appropriate secondary antibodies (Mouse sections: CF488A donkey anti-rabbit [1:2000; Sigma, SAB4600036]; human sections: A647 donkey anti-mouse [1:500; Molecular probes, A31571]). Sections were then washed 3 times 10 min with PBS and stained with 16 mg/L TR (mouse sections only) in PBS for 5 min. After further washings with PBS to eliminate TR excess, the sections were counter stained for DAPI and mounted using ProLong Diamond antifade mountant (Invitrogen, P36961). Before DAPI staining, human brain sections were treated with True Black to further quench autofluorescent lipofuscins [42]. Slides were imaged on an Imager Z1 microscope (Zeiss) equipped with an AxioCam MR R3 camera. Mouse brain images were acquired with a 20X/0.50 M27 Zeiss Plan-Neofluar air objective; 11 images (corresponding to 10-µm-thick optical sections) were acquired. Human brain images were acquired with a ×40 Plan Apochromat 1.4 NA oil DIC oil objective; 21 images (corresponding to 5-µm-thick optical sections) were acquired. Detection of the green fluorescence channel (P2RY12 mRNA expression) was obfuscated by the autofluorescence.  To improve the image quality, detection of P2RY12 mRNA expression was performed using the “Spots” plugin of the IMARIS software with detection of spots of at least 1 µm in diameter [43]. Therefore images shown in the green channel are reconstituted to enable visualization without potential artifacts of autofluorescence.

## Results

**Fig. 1:**
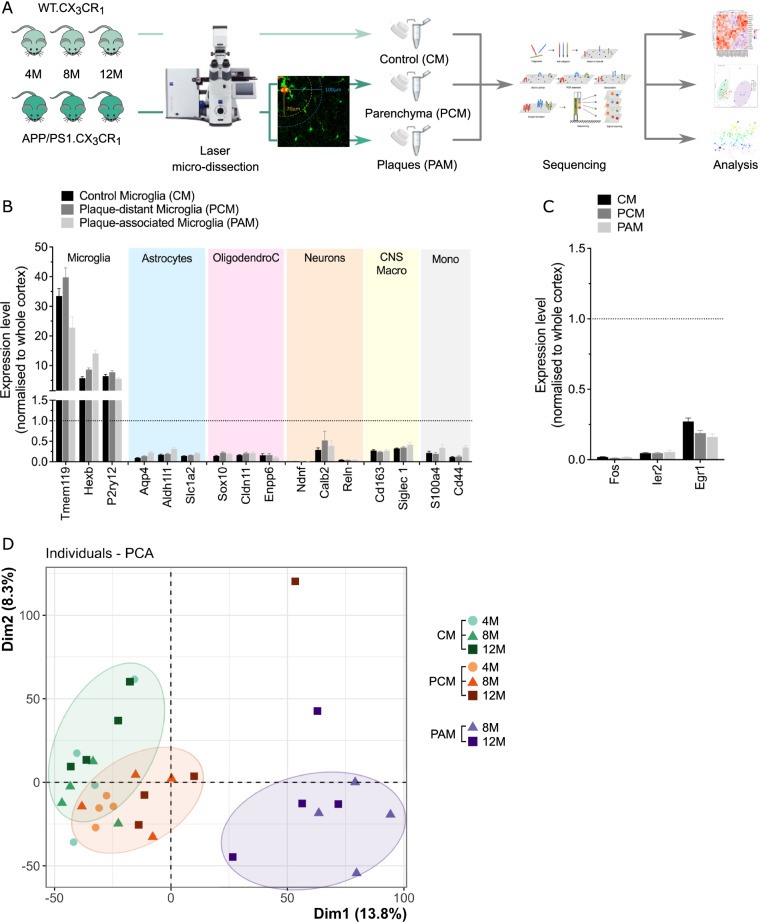
Study design and validation of the approach. **A** Schematic representation of the study design. **B** Gene expression of cell-type markers for microglia, astrocytes, oligodendrocytes, neurons and peripheral immune cells in laser captured control microglia from CX3CR1^+/eGFP^ mice (CM, black), plaque-distant microglia (PCM, grey) or plaque-associated microglia (PAM, light grey) from APP/PS1^Tg/0^:CX3CR1^+/eGFP^ mice. Gene expression is normalized to housekeeping genes expressions and set at 1 in whole cortex homogenates. **C** Gene expression of immediate early genes in the microglial sub populations. Gene expression is normalized to housekeeping genes expression and set at 1 in whole cortex homogenates. **D** Principal component analysis (PCA) of gene expression in the different microglia samples, based on the 13,923 expressed genes

We analyzed the repertoire of genes expressed in cortical microglia from both control and AD-mice (Fig. 1A) by combining laser capture microdissection (LCM) and RNA-seq approaches in transgenic mice expressing eGFP under the control of the CX3CR1 promotor [25, 26]. Moderate tissue fixation preserves fluorescence but alters the quantity and quality of recovered RNA, whereas eGFP fluorescence is generally low in fresh unfixed brain samples [44]. To overcome these issues, we developed a specific protocol based on tissue preservation by sucrose perfusion and immersion, rapid freezing in − 40 °C isopentane, cryo-sectioning and dehydration, which allows eGFP fluorescence preservation, amyloid plaque staining as well recovery of RNA in good quality and quantity (Additional file 1: Fig. S1A).

We extracted total RNA from 400 to 600 microglia per animal per experimental condition (Additional file 14: Table S2) and performed mRNA sequencing. We detected 13,923 expressed genes (Additional file 15: Table S3), and compared the expression of 133 randomly selected ones (i.e., exhibiting low, medium or high expression levels) in FACs-sorted microglia [37]. Linear regression revealed a significant correlation between gene expression levels in LCM and FACS-isolated microglia (*r* = 0.697, *p* < 0.0001), demonstrating that our data are consistent with previously published microglia gene expression profiles (Additional file 1: Fig. S1B).

### Isolation of microglia from mouse brain tissue with preservation of spatial information

Cell isolation through LCM allows preservation of spatial information, but is subject to cross-contamination by surrounding cells whose processes may by be captured together with the cell of interest. To assess the degree of microglial enrichment, we evaluated the expression levels of specific microglial, astrocytic, oligodendrocytic and neuronal genes in the different LCM samples. Figure 1B shows that microglia-specific genes are about 10 times enriched in the LCM samples compared to the whole cortex tissue, whereas, reversely, other glial cells and neurons specific genes are strongly depleted. In CX3CR1^+/eGFP^ mice, eGFP is expressed in all myeloid cells including infiltrating monocytes that can penetrate brain parenchyma in pathological conditions [45]. To assess the possibility that our LCM samples could be contaminated by CNS macrophages and/or infiltrating monocytes, we analyzed the expression of CNS macrophages and peripheral monocyte/macrophage specific genes (Fig. 1B). We demonstrated that the expressions of *Cd163* & Siglec1, and *S100a4* & Cd44 which are, respectively, markers of CNS macrophages and circulating monocytes were also depleted in the LCM samples.

An important drawback of cell isolation is the intrinsic cellular activation induced by generating single-cell suspension. This is particularly true for microglia which are inherently reactive cells, for which it has been shown that FACS sorting alters the analysis of the disease-induced transcriptomic changes [20, 46]. In contrast to FACS, in LCM, cells are isolated from their environment after dehydration, which prevents cell reaction as shown by low expression of immediate early genes in the LCM-isolated microglia (Fig. 1C).

The reproducibility of our data was demonstrated by the strong correlation between the biological replicates (Additional file 1: Fig. S1C). In addition, principal component analysis (PCA) on mRNA expression profiles allowed good discrimination between the different microglia subtypes (Fig. 1D). In particular, PAM clearly separate from both CM and PCM, but remarkably we show that the PCM also separate from the CM. However, this analysis did not allow further separation of the samples by age within each sub-population. We then examined, in the different subpopulations, gene expression for markers of previously identified microglia gene signatures, namely markers of homeostatic microglia (*P2ry12 & Tmem119*, Additional file 2: Fig. S2A), disease-associated microglia (DAM; *Apoe & Ctsd*, Additional file 2: Fig. S2B (11)), activated-response microglia (ARM; *Cst7, H2-ab1*, *Itgax*, Additional file 2: Fig. S2C (13)) and interferon-response microglia (IRM; *Ifit2, Ifit3 & Oasl2*, Additional file 2: Fig. S2D [13]). Our results showed in contrast to previous studies, homeostatic microglial gene expression levels remained stable in CM and PCM, and appeared to decrease only slightly in late stage PAM (Additional file 2: Fig. S2A and Table S4). Interestingly, our results revealed that DAM, ARM and IRM markers are also up-regulated in PCM with their expression increasing in an age-dependent manner (Additional file 2: Fig. S2B–D). Notably, expression of DAM and ARM markers remained stable across disease stages in PAM, whereas that of IRM appeared to increase in older PAM (Additional file 2: Fig. S2C, D).

Having established this protocol allows isolation of spatially distinct sub populations of microglia with minimal intrinsic perturbation and sufficient enrichment, we conducted specific contrast analyses to identify DEGs between different sub populations. We then performed bioinformatic/biostatistic analyses to infer the pathophysiological role of the different microglia subpopulations in AD progression. The workflow for data analysis is presented in Additional file 3: Fig. S3.

### Biological functions and master gene regulators in PAM

In the APP^swe^/PS1^dE9^ model, dense amyloid plaques begin to appear in cortical areas at about 4-months of age [26]. However, at this age, plaques are very sparse and it was not technically feasible to isolate PAM. Thus, to identify gene deregulation in PAM versus CM microglia, we restricted the analysis to 8-mo and 12-mo samples.

**Fig. 2:**
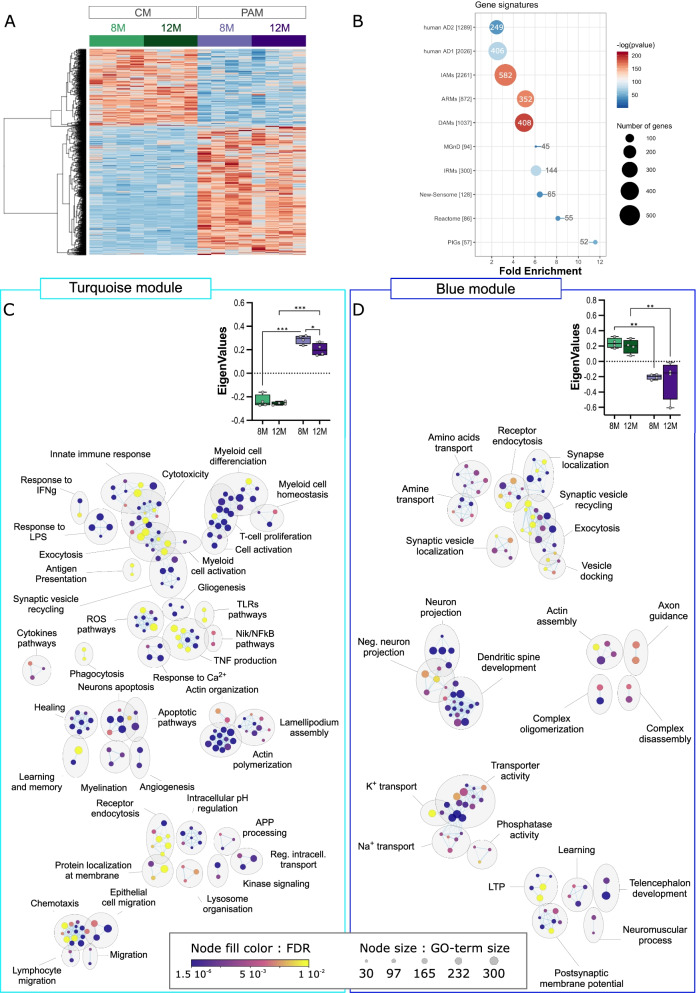
Microglial gene expression remodeling in plaques associated microglia. **A** Heatmap of differentially expressed genes in PAM (violets) versus CM (greens) along aging. The scaled expression value (row Z score) is shown in a blue–red color scheme with red indicating higher expression, and blue lower expression. The full data are available in Additional file 17: Table S5A. **B** Enrichment in DEGs in different gene signatures. The size and the color of each dot are, respectively, proportional to the number of DEGs and the adjusted *p*-value. The number of DEGs is also indicated in or at proximity of the dot. **C**, **D** Cytoscape representation of deregulated GO terms (“Biological Process” category) in the turquoise (C) and blue (D) gene modules. The full data are available in Additional file 17: Table S5B, C. Each dot represents a deregulated GO-Term; their size and color are, respectively, proportional to the number of genes in the GO-term and the enrichment adjusted p-value. GO-Terms are grouped into categories using the Autoannotate Cytoscape app. Insets: Boxplots of eigengene values for each sample in the respective modules (see also Additional file 4: Fig. S4)

PCA revealed a clear distinction between CM and PAM, and statistical analysis identified 1851 DEGs (false discovery rate (FDR) < 0.05), about two-thirds of which were up-regulated (Fig. 2A and data not-shown). Previous studies established mouse microglia reaction signatures in different pathological conditions. This includes the DAM, ARM and IRM signatures previously mentioned [11, 13], but also signatures for inflammatory-associated microglia (IAM) which represent a large set of DEGs in inflammatory conditions [38] and for the reactome a smaller set of 86 genes deregulated in different acute and neurodegenerative conditions [37]. More recently, using a snRNAseq approach, Gerrits et al. [14] identified two human microglia reaction signatures associated with AD pathology. The abundance of the AD1 subpopulation correlated with tissue Aβ load, whereas that of AD2 subpopulation better correlated with samples with overt Tau pathology. Figure 2B shows that, compared to control microglia, PAM DEGs were significantly enriched for these different pathological microglia gene signatures. The sensome gene signature represents a set of membrane-associated proteins and receptors that are selectively expressed in microglia and that help them sense changes in their environment [36] (refined in [37]). This signature was also significantly affected in PAM. Using a spatial transcriptomic approach, Chen et al. recently identified a plaque-induced gene (PIG) network mainly involving microglial and astroglial genes [21]. As expected, PAM signature was strongly enriched with PIGs genes, as 52 of the 57 PIGs genes were deregulated in PAM (Fig. 2B).

Genes with similar expression patterns (e.g., co-expression) are likely to have similar functions and can be grouped into modules by WGCNA [34]. We performed WGCNA analysis to identify gene modules among the DEGs, and GO-based enrichment analyses to extract the hypothetical biological functions for each of these modules (see Additional file 3: Fig. S3 and Materials and methods for details). Among the 1851 DEGs, we identified two distinct modules significantly correlated with the *Microglia-subtype* trait. The largest module (i.e., turquoise module) included 1639 genes mainly up-regulated in PAM (Fig. 2C and Additional file 17: Table S5A). These genes were primarily associated with inflammation related biological processes, including *Cell activation & proliferation*, *Immune response*, *Cytotoxicity*, *Exocytosis*, *Chemotaxism*, *Antigen presentation*, etc. (Fig. 2C; Additional file 17: Table S5B). Alterations in *Cell morphology* was another significantly affected biological function. The second module (i.e., blue module) was smaller and contained 212 genes mainly down-regulated in PAM (Fig. 2D; Additional file 17: Table S5A). This module mainly related to *Synaptic transmission* associated biological processes (Fig. 2D; Additional file 17: Table S5C).

In gene networks or subnetworks, hub genes (i.e., most highly connected genes) represent master regulators that are likely to play essential roles in controlling the biological response. We used specific applications in Cytoscape (see Materials and methods) to first construct the genes’ network of the two WGCNA modules, and second to identify the most connected subnetworks and their potential hub genes. In the turquoise module, the 1074 most highly connected genes were separated into subnetworks using the MCC cluster tool. The ten larger clusters are detailed in Additional file 18: Table S6, with hub genes highlighted in dark green. The three larger subnetworks are also shown in Additional file 4: Fig. S4B–D, with hub genes in yellow. In the largest subnetwork, *App* and *Penk* which are, respectively, up- and down-regulated in PAM appeared to play orchestrating and redundant roles for controlling chemotaxis and endopeptidase activities (Additional file 4: Fig. S4B). Importantly, we confirmed that *App* detected in our experimental conditions corresponds to mouse *App* and not *hAPPswe,* ensuring up-regulation was not due to contamination by adjacent neurons. The second subnetwork included genes that control cell shape and antigen processing. Hub genes of this network were the GTPases *Rac1*, *Rhoa* and *Rhog* which belong to, respectively, the Ras and Rho super-families (Additional file 4: Fig. S4C). *Rab5c* is another small GTPase involved in controlling receptors endocytosis, vesicle trafficking, and endo-lysosomal pathways [47] and which played a central role in the third largest subnetwork (Additional file 4: Fig. S4D). In the blue module, we identified a single network of 48 highly connected genes (Additional file 4: Fig. S4F). This gene network was associated with control of the synaptic vesicle cycle (Additional file 18: Table S6); hub genes were *Syt1*, *Vamp2* and *Snap25* which represent key proteins for neurotransmitter release.

Next, we addressed the question of the extent to which age affected the transcriptomic changes observed in PAM. To meet this goal, RNA-seq data from the 8-mo and 12-mo samples were reanalyzed using a Generalized Linear Model (GLM) model, with *Microglia-subtype* (PAM vs CM) and *Age* (8-mo vs 12-mo) as between samples’ factors (Additional file 5: Fig. S5). Thus, we identified 723 DEG in the *Microglia-subtype:Age* interaction (raw *p*-value < 0.05). Among them, we restricted our analysis to the 179 genes that were significantly deregulated in PAM vs CM (Additional file 5: Fig. S5A). WGCNA analysis further identified three genes modules (turquoise, blue and brown) that were significantly correlated with the *Microglia-subtype* trait. The largest module (i.e., turquoise module) included 96 genes that related to *i) Wound healing*, *Cell projection organization* and *PKB signaling* biological processes; *ii)* *Bacterial invasion* and *Phagosome* KEGG pathways and *iii)* *Ephrin signaling* KEGG pathways (Additional file 5: Fig. S5C)*.* Interestingly, these genes were more strongly over-expressed in 8-mo versus 12-mo PAM and were slightly over-expressed in 12-mo CM suggesting that the normally occurring overexpression of these genes during normal ageing was accelerated in PAM (Additional file 5: Fig. S5B). The blue module regrouped genes that were mostly down-regulated in PAM and tended to be less expressed/more down-regulated in 8-mo PAM. Moreover, these genes were also down-regulated in 12-mo CM compared to 8-mo CM. Genes of this module did not relate to any specific GO biological processes, but were associated with *MAP kinases*, *ErbB signaling* and *mitophagy* KEGG pathways (Additional file 5: Fig. S5C). Finally, genes of the brown module are up-regulated in PAM but down-regulated in older CM (Additional file 5: Fig. S5B). These genes are associated to *hypoxia* related and *cell adhesion* pathways (Additional file 5: Fig. S5C).

Overall, our comparison of PAM versus CM in the APP/PS1 model shows that PAM exhibit profound transcriptomic changes which drive an increased inflammatory reaction, support morphological changes and contribute to the degradation of synaptic support functions. Although location at the proximity of Aβ-plaques is the most important driver for transcriptomic changes, *Age* contributes, but to a lesser extent, to the observed alterations. Notably, in aged PCM, the expression profiles of the 1851 DEGs, were intermediate between what was found in CM and in PAM (Additional file 8: Fig. S8A).

### Biological functions and master gene regulators in PCM

Because amyloid plaques relate to one of the most prominent features of the disease, studies on the role of microglia in AD have often focused on PAM. Quite the reverse, PCM whose morphology is very similar to that of CM are generally less well studied (Additional file 6: Fig. S6A). However, these cells are also part of the pathological environment and we reasoned that they are likely to also contribute to the disease progression.

**Fig. 3:**
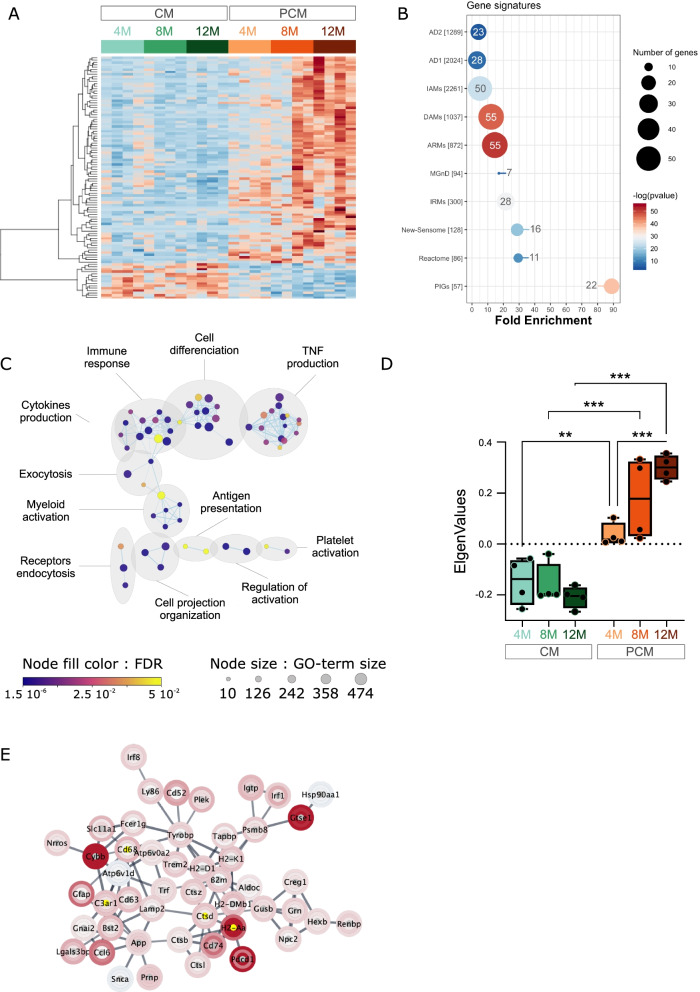
Microglial gene expression remodeling in plaque-distant AD microglia. (A) Heatmap of differentially expressed genes in PCM (oranges) versus CM (greens) along aging. The scaled expression value (row Z score) is shown in a blue–red color scheme with red indicating higher expression, and blue lower expression. The full data are available in Additional file 19: Table S7A. (B) Enrichment in DEGs in different gene signatures. The size and the color of each dot are, respectively, proportional to the number of DEGs and the adjusted p-value. The number of DEGs is also indicated in or at proximity of the dot. (C) Cytoscape representation of deregulated GO terms (“Biological Process” category) in PCM versus CM DEGs. The full data are available in Additional file 19: Table S7B. Each dot represents a deregulated GO-Term; their size and color are, respectively, proportional to the number of genes in the GO-term and the enrichment adjusted p-value. GO-Terms are grouped into categories using the Auto-annotate Cytoscape app. (D) Boxplots of eigengene values for each sample in the most abundant WGCNA module of PCM versus CM DEGs (91 genes). Statistical analyses: 2-ways ANOVA with *Microglia subtype* and *Age* as between subjects’ factors. *Subtype* *p* < 0.001; *Subtype:Age* *p* < 0.01. FDR corrected post hoc tests; **p < 0.01, ***p < 0.001. (E) Cytoscape network visualization of highly connected genes in PCM versus CM DEGs. Log_2_ expression ratio between PCM and CM are mapped to the nodes using a blue–white–red gradient with red indicating higher expression in PCM, and blue lower expression. The inner, middle and outer rings represent log-ratio in, respectively, the 4-, 8- and 12-mo samples. Yellow nodes depict hub genes within the network

To investigate whether specific biological functions were altered in PCM versus control CM, we identified genes significantly deregulated between the two conditions irrespective of age. PCA discriminated PCM from CM according to the second dimension (Additional file 6: Fig. S6B), and statistical analysis identified 102 DEGs (FDR < 0.05), the great majority (87/102) of which were up-regulated (Fig. 3A, Additional file 19: Table S7A). Interestingly, as for PAM, deregulated PCM DEGs were very significantly enriched for the different murine and human pathological microglia gene signatures (i.e., IAM, DAM, ARM, MGnD, IRM, AD1, AD2 signatures), the reactome and the sensome signatures (Fig. 3B). More surprisingly, PCM’s DEGs were also highly enriched for PIG gene network. However, this may be explained by the fact that in Chen et al. [21] study, amyloid load was quantified based on 6E10 immunostaining which labels more diffuse Aβ plaques. In agreement, we showed approximatively one-tenth of 6E10 + plaques are not labeled by TR (Additional file 12: Fig. S12B, C). GO analyses also revealed that these genes are associated with immune-related functions, including *Tumor Necrosis Factor* (*TNF)* and *Cytokine production*, *Immune response* and *Antigen presentation* (Fig. 3C; Additional file 19: Table S7B). Cellular reaction in this microglia subtype was also demonstrated by deregulation of functions linked to *Cell differentiation* and *Myeloid activation*. Among those DEGs, WGCNA analysis identified 2 distinct modules of co-deregulated genes (Table S7A). The largest one contained the vast majority of the DEGs (91/102) and corresponded to genes that showed an age-dependent up-regulation in PCM (Fig. 3D). The second module was limited to only 7 genes, including *App* and could not be related to a specific biological function (data not shown). On the other hand, gene network analysis identified a cluster of 49 highly connected genes that are strongly associated with the *Lysosome* (*p* = 4.1 × 10^–11^), the *Antigen processing and pre*sentation (*p* = 2.4 × 10^–10^), and the *Phagosome* (*p* = 7.4 × 10^–6^) KEGG pathways (Fig. 3E). *Cd68*, *Ctsd*, *H2-aa* and *C3ar1* represented hub genes within this network.

As shown in Fig. 3A, gene expression changes were quite variable in PCM with a general trend for higher deregulation in microglia isolated from older mice. Additionally, although the expression changes were more similar within 4-mo and 12-mo samples, the inter-individual variation appeared greater in microglia isolated at in 8-mo mice. To address whether age affected the transcriptomic changes observed in PCM, we first identified 1334 genes deregulated in PCM versus CM (raw *p*-value < 0.05), and then searched among them which ones are also deregulated in the *Microglia-subtype*:*Age* interaction (raw *p*-value < 0.05) (Additional file 6: Fig. S6C). We thus identified 595 genes whose expression changed in PCM in an age-dependent manner. WGCNA analysis further identified two gene modules. The largest one (turquoise module) was significantly correlated with the *Microglia-subtype* factor. Genes of this module were up-regulated in the intermediate and late stage of the disease (Additional file 6: Fig. S6D, upper panel) and related to inflammatory processes, notably *Cytokine production*, *Antigen presentation*, *Myeloid cell activation* and the *Phagosome* KEGG pathway (Additional file 6: Fig. S6E). Interestingly, these genes showed opposite regulation in CM being less expressed in 12-mo compared to younger cells. The second module (blue module) was significantly correlated with the *Age* factor and contained genes whose expression were down-regulated in an age-dependent manner specifically in PCM (Additional file 6: Fig. S6D, lower panel). Genes of the blue module related to *Lipid oxidation*, *Organelle transport* and *Synaptic* transmission biological processes (Additional file 6: Fig. S6E).

Overall, results demonstrated that although PCM are not associated with Aβ plaques, and display homeostatic-like morphology, they exhibit age-dependent transcriptome alterations. These alterations are associated with important microglial functions that are typical of microglial reaction. Of note, the great majority of the 102 genes dysregulated in PCM were also dysregulated in PAM and showed increased dysregulation compared to that measured in PCM (Additional file 8: Fig. S8B).

### To what extent do PAM and PCM differ?

**Fig. 4:**
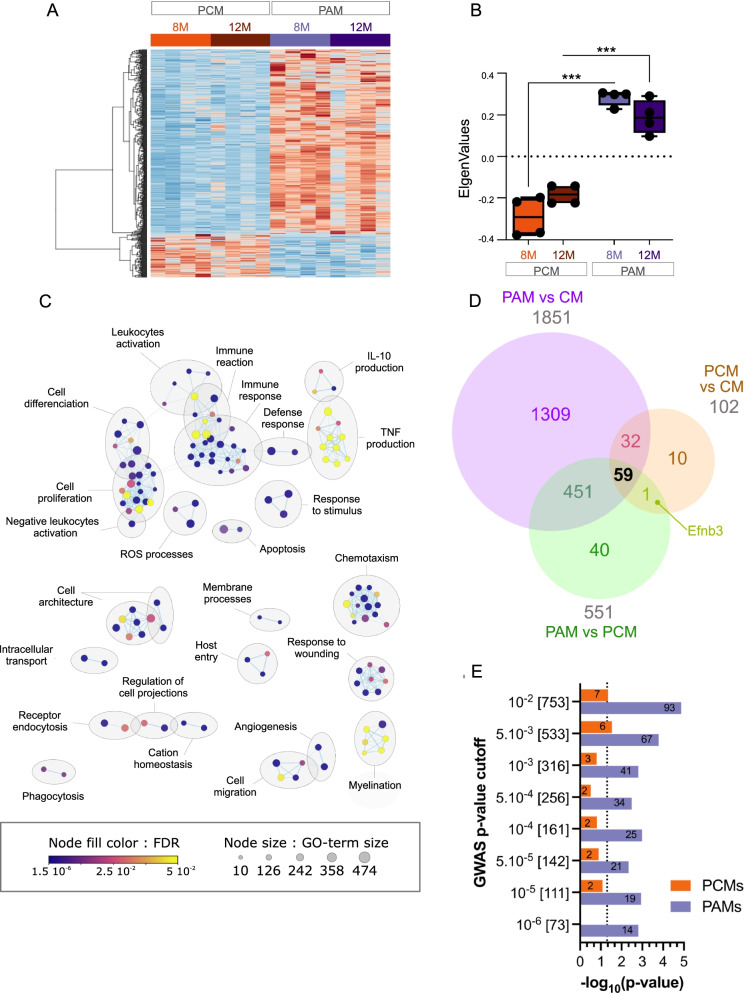
Microglial gene expression remodeling between plaques-associated and plaques-distant AD microglia. **A** Heatmap of differentially expressed genes in PAM (violet) versus PCM (orange) along aging. The scaled expression value (row Z score) is shown in a blue–red color scheme with red indicating higher expression, and blue lower expression. The full data are available in Additional file 20: Table S8A. **B** Boxplots of Eigengene values for each sample in the most abundant WGCNA module of PAM versus PCM DEGs (497 genes). Statistical analyses: 2-ways ANOVA with *Microglia subtype* and *Age* as between subjects’ factors. *Subtype* *p* < 0.001; *Subtype:Age* *p* < 0.05. FDR corrected post hoc tests; ***p < 0.001. **C** Cytoscape representation of deregulated GO terms (“Biological Process” category) in the most abundant module of PAM versus PCM DEGs (497 genes). The full data are available in Table S8B. **D** Venn diagram showing the overlap between DEGs in PAM versus CM (FDR < 0.05, violet circle), PCM versus CM (FDR < 0.05, orange circle), and PAM versus PCM (FDR < 0.05, green circle). (E) Bar plot showing the significance (− log10(*p*-value)) of enrichment of AD GWAS genes in PAM and PCM DEGs. Different AD GWAS sets corresponding to different p-value cutoffs (Marioni al., 2018), were tested. The numbers under brackets indicate the number of ortholog genes at the cutoff, and the numbers in columns, the numbers of PAM or PCM DEG GWAS genes

To further investigate the extent to which PAM and PCM differ at the transcriptomic level, we searched for genes significantly deregulated between the two microglia subtypes. Considering both the 8-mo and 12-mo samples, we identified 551 DEGs (FDR < 0.05), of which 80% (446/551) were up-regulated in both PAM vs PCM (Fig. 4A). WGCNA analysis identified a single module of 497 genes, which was significantly correlated with the *Microglia-subtype* trait (*r* = 0.95; *p* < 2.10^–8^) and more highly expressed in PAM (Fig. 4B, Additional file 20: Table S8A). These 497 genes were associated with inflammation related biological processes (Fig. 4C; Additional file 20: Table S8B) and, at least in part, overlapped with those deregulated in PCM vs CM, indicating that PCM present an intermediate reactive state between CM and PAM. However, some biological functions were specific to PAM, including *Chemotaxism*, *Cell proliferation*, *Cell architecture* and *ROS production*. By comparing the three lists of DEGs (i.e., PAM vs CM, PCM vs CM and PAM vs PCM), we also identified 11 genes that were deregulated in PCM only (Fig. 4D). Of these genes, eight (i.e., Zfp607, Zfp808, Abcc9, Tstd2, Efnb3, Wscd1, Trp53inp2 and Emc2) showed significantly greater expression in PCM compared to CM, whereas in PAM their expression was either not different or lower than in CM (Additional file 21: Table S9A). This latter result suggests that PCM are also engaged in specific functions compared to PAM. This small panel of genes could not be associated to any specific biological processes (not shown). Despite this limitation, among these 8 genes, *Efnb3*, which codes for a transmembrane ligand for Ephrin receptors, was significantly more expressed in PCM compared to either CM or PAM (Additional file 7: Fig. S7E).

Among the genes deregulated in PAM versus PCM, we identified 96 genes changed in the *Microglia-subtype*:*Age* interaction (raw *p*-value < 0.05) (Additional file 7: Fig. S7A). WGCNA analysis refined this list to 91 co-expressed genes that were more highly expressed in PAM compared to PCM. These genes were enriched for biological processes associated with *Actin filament organization*, *Cell migration and differentiation*, *Peptidase activity* (Additional file 7: Fig. S7B and Additional file 22: Table S10) and for the KEGG *Chemokine signaling* pathway (Additional file 22: Table S10). They showed opposite age-dependent regulation in PCM and PAM, and thus globally appeared less up-regulated in 12-mo PAM (Additional file 7: Fig. S7C, D).

These results indicate that although PAM and PCM share common signaling pathways, they are also engaged in specific biological functions. These cell-type specific data also reveal different age-dependent regulations in PCM and PAM. Further, in CM, the expression profiles of the 551 DEGs reflected a similarity with PCM (Additional file 8: Fig. S8A).

To further explore the relative contribution of PCM and PAM to AD, we tested whether AD risk genes were enriched among the PAM and PCM DEGs. We used a list of curated genes from an extensive GWAS study, converting the human ID genes to the respective murine orthologs [48]. Recent studies on polygenic risk scores have shown that genes with even small significance in GWAS carry information with regard to AD [49]. Accordingly, we tested for enrichment in GWAS genes at different cutoffs (Fig. 4E). At all cutoffs (p-values ranging from 10^–6^ to 10^–2^), PAM DEGs were significantly enriched for GWAS-associated AD genes suggesting that PAM play a key role in AD pathogenesis. In contrast, PCM DEGs were significantly enriched for GWAS AD genes only at the lowest cutoffs. This suggests that PCM may contribute to AD pathogenesis, albeit to a lower extent than PAM.

### Validation in mouse and human brain tissue

**Fig. 5:**
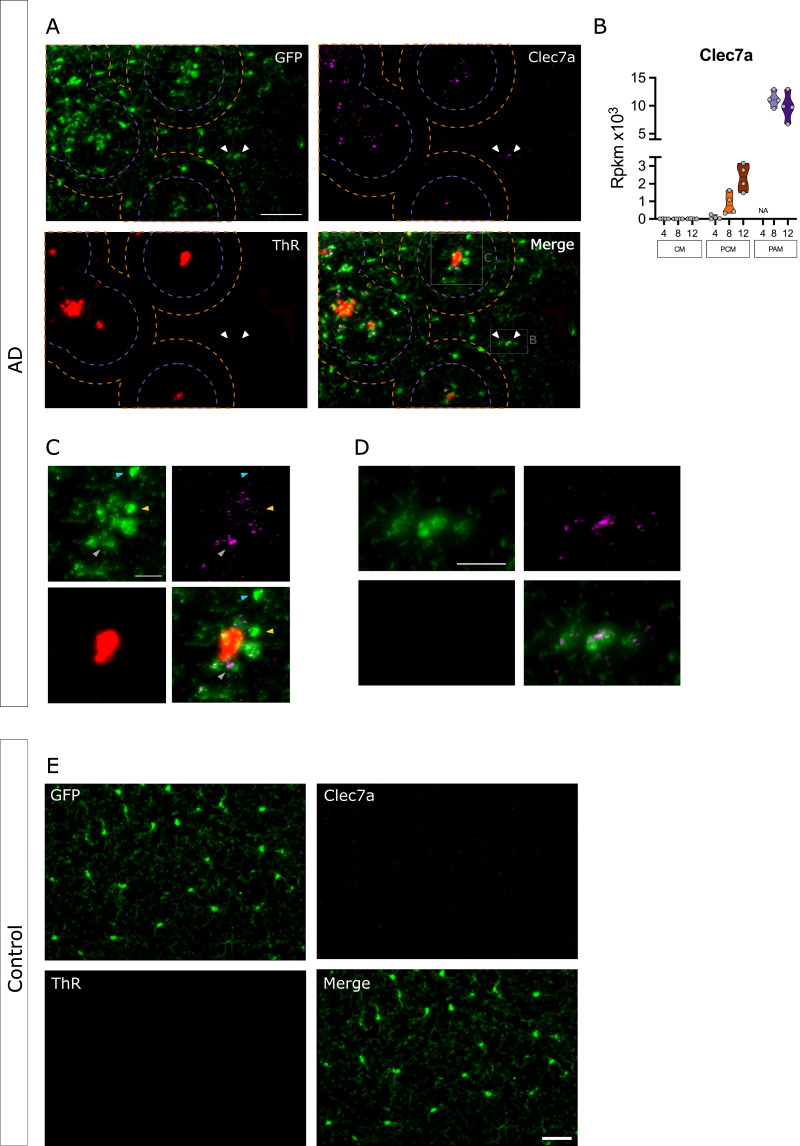
Clec7a mRNA expression in control (CM); plaques-distant (PCM) and plaque-associated (PAM) microglia. Microglia detection (GFP, green), Thiazine Red (TR) staining for amyloid plaques (red) and *Cst7* mRNA expression (purple) were combined on brain slices in **A** APP/PS1^Tg/0^: CX3CR1^+/eGFP^ or **E** CX3CR1^+/eGFP^ mouse. **A** 12-mo APP/PS1^Tg/0^:CX3CR1^+/eGFP^ mouse cortical field exhibiting amyloid plaques. Plaque-associated microglia (PAM) are located within 70 µm (purple dotted circle) of the plaque epicenter; while plaque-distant microglia (PCM) are located farther than 100 µm (orange dotted circle) from the plaque epicenter. *Clec7a* mRNA expression is absent from control microglia (**E**) but can be evidenced in both plaque-associated (**A**, **C**) and plaque-distant (**A**, **D**) microglia. White arrowheads point to *Clec7a* positive plaque-distant microglia. **B** Pseudocounts of *Clec7a* expression in CM (greens), PCM (oranges) and PAM (violets) along ages. **C** Zoomed region around TR+ plaque showing *Clec7a* overexpression in PAM. Note the heterogeneity for *Clec7a* expression among PAM, blue arrowhead for no expressing microglia, yellow arrowhead for low expressing microglia, grey arrowhead for high expressing microglia. **D** Zoomed region of *Clec7a* positive plaque-distant microglia. Scale bar 20 µm

**Fig. 6:**
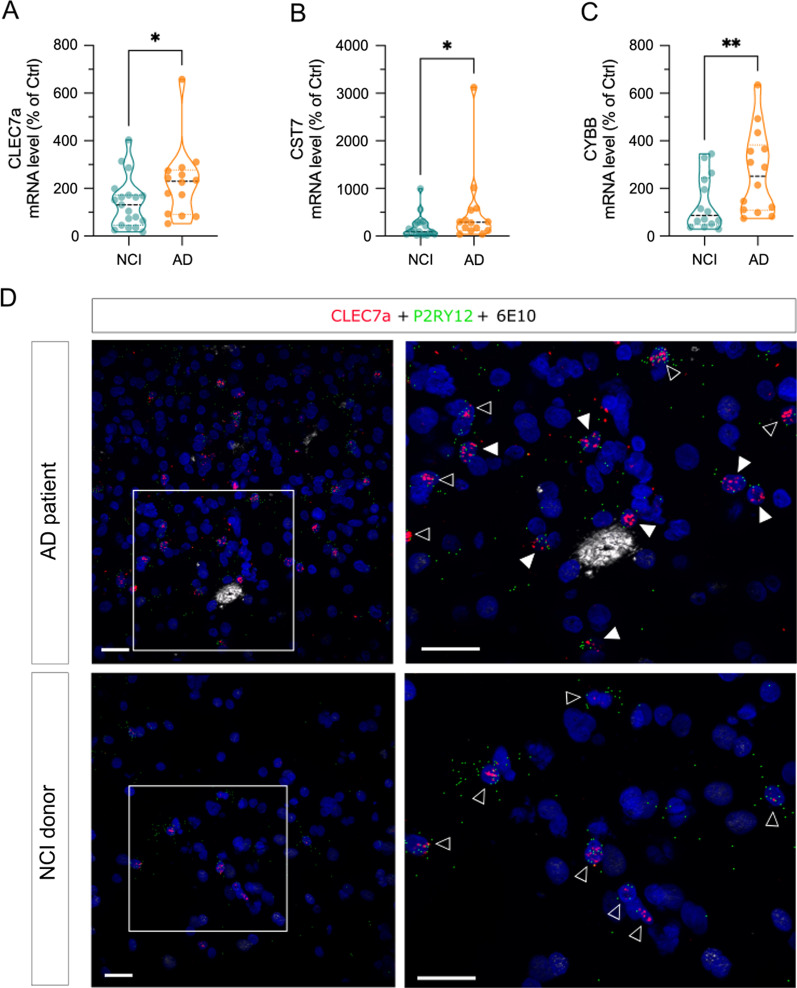
CLEC7a, CST7 and CYBB mRNA expression in the prefrontal cortex of AD patient and NCI subjects. **A**–**C** Changes in mRNA expression of CLEC7a (**A**), CST7 (**B**) and CYBB (**C**) were measured by qPCR in samples from the prefrontal cortex of AD and NCI subjects; **D** microglia detection (P2RY12 mRNA, green, reconstituted image), 6E10 staining for amyloid plaques (white) and *CLEC7A* mRNA expression (red) were combined on slices from BA9-10 of AD and NCI subjects. Moderate level of *CLEC7A* is observed in both plaque-associated and plaque-distant microglia of AD patients (representative image from a 83 years old female). Overall lower level of mRNA expression is observed in microglia from NCI subjects (representative image from a 43 years old male). Right panels show zoomed regions; plain white arrowheads point to *CLEC7A-*positive plaque-associated microglia and open arrowheads *CLEC7A-*positive plaque-distant microglia. Scale bar 30 µm

Laser capture microdissection can be used to isolate discrete cells from complex environments while preserving spatial information, however, the cell populations obtained by this method are not pure. To validate the cellular and spatial localization of the DEGs, we selected 3 genes (*Cst7*, *Cybb* and *Clec7a*) that show significant deregulation in both PAM and PCM and performed single-molecule fluorescence in situ hybridization (smFISH) with specific RNAScope probes against these targets (Fig. 5 & Additional file 10: Fig. S10, Additional file 11: Fig. S11). smFISH was coupled with immunofluorescent detection of microglia using anti-GFP antibody and amyloid plaques using Thiazin red staining (Fig. 5 and Additional file 10: Fig. S10, Additional file 11: Fig. S11). In agreement with the robust up-regulation of their mRNAs in PAM, *Cst7*, *Clec7a* and *Cybb* signals strongly colocalized in microglia associated with amyloid plaques (Fig. 5A,C [*Clec7a*]; S10-A,C [*Cst7*] and S11-A,C [*Cybb*]). However, weaker but positive RNAscope signals for *Cst7*, *Clec7a* and *Cybb* were also observed in microglia located further than 70 µm away from plaques (Fig. 5A,C [*Clec7a*]; S10-A,C [*Cst7*] and S11-A,C [*Cybb*]). We also observed a pronounced heterogeneity in the level of expression of these mRNAs in microglia independent of their association with Aβ plaques. Indeed, some microglia expressed high levels of target mRNA, while neighboring microglia expressed little or no mRNA at all (Fig. 5C; Additional file 10: S10C and Additional file 11: Fig. S11C; see arrowheads). Employing an independant qPCR approach, we also validated increased expression for these three genes in samples from the postmortem prefrontal cortex of AD patients compared to age-matched NCI subjects (Fig. 6A). qPCR is a global approach that does not distinguish expression in PAM and PCM. To validate the cellular and spatial localization of these DEGs we performed smFISH on tissue sections from BA9-10 of AD and NCI subjects. We focused on CLEC7a as this gene is among the most prominent up-regulated ones (Additional file 19: Table S7). Using P2RY12 specific RNAscope probe to detect microglia and 6E10 to label Aβ deposits, we validated the expression of CLEC7A in both PAM and PCM (Fig. 6B), confirming microglia gene expression findings from the murine model in bona fide human AD brain.

## Discussion

Microglia reaction in AD was initially evidenced at the genomic level using bulk RNA-seq studies performed on purified microglia [6, 7]. The molecular heterogeneity of microglia in this pathological context was then studied using top-down approaches (typically scRNA-seq). With these approaches, microglia subtypes are first identified based on transcriptomic similarities, their potential functions are then inferred based on gene-ontology analyses, and finally their location in the tissue are assessed retrospectively based on the expression of a handful of markers [11–13, 50]. Other studies isolated PAM and/or PCM using FACS, relying on specific markers that were shown to be, on average, up-regulated in PAM. These studies then performed thorough characterization of the positive and negative populations [17, 19].

Herein, to decrypt alterations in microglial cells that are or not associated to Aβ-plaques, we used an alternate strategy based on an unbiased bottom-up approach. We combined laser capture microdissection and RNA-seq to study transcriptome remodeling in these two spatially defined microglia subpopulations. Further, we investigated the evolution of these alterations during the progression of the disease, from the early stage, when the plaques barely form, to the late stage, when Aβ-plaque load is prominent throughout the cortex. We confirmed that dense amyloid plaques drive striking transcriptomic alterations, leading to reactive microglia that display strong inflammatory phenotypes and are less supportive to neuronal functions. Our study also provides the first in-depth characterization of spatially identified plaque-distant microglia (PCM), highlighting that although this microglia subtype does not show major morphological alterations, it exhibits, from the early stages, an increased inflammatory phenotype that progresses with the development of the pathology. Thus, our study reveals that PAM and PCM are both involved in AD progression, and engaged in functions which are only partially overlapping.

### Combination of LCM and RNA-seq provide a unique way to decipher the respective roles of discrete microglia subpopulations

We have previously shown that *Cx3cr1* haplodeficiency does not alter disease progression in the APP/PS1 model [26]. Here we demonstrate that quick TR staining and laser microdissection procedures preserve RNA quality. By isolating no less than 1600–2000 microglia cells from 4 mice per experimental conditions, i.e., approximatively 400 cells per mouse in each experimental condition (Additional file 14: Table S2), we were able to accurately analyze the transcriptome of spatially distinct microglial populations.

Although LCM does not match the purity fidelity of FACS, our data reveal  that the preponderance of cells analyzed are microglia. First, expression of different brain cell-specific markers revealed a tenfold enrichment of microglial markers with parallel depletion in astrocyte, neuronal and oligodendrocyte markers. Second, in situ hybridization of specific genes of interest confirmed their microglial localization. In CX3CR1^+/gfp^ mice, all myeloid cells express GFP, thus we cannot exclude that some of the microdissected cells are in fact infiltrated monocytes or peri-vascular macrophages. However, contamination by these specific cell types is likely to be low since they tend to express GFP at lower level [51], whereas we preferentially selected high GFP expressing cells for microdissection. Consistent with a strong enrichment of microglia in the LCM-isolated cells, we showed a high correlation between gene expression in LCM and FACs-isolated GFP+ cells from CX3CR1^+/gfp^ mice. We also showed reduced expression of macrophage and monocyte markers in the LCM-isolated cells. This finding is consistent with  *i)* the transcriptional remodeling observed in tissue surrounding Aβ-plaques [22] and   *ii)* recent studies in both mouse and human tissues showing that microglia are the only myeloid cells present at the vicinity of Aβ deposits [52, 53], further endorsing the extremely high probability that the cells we analyzed were indeed microglia.

To date, the majority of microglial gene interrogations in AD has come from RNA-seq [6, 7] and scRNA-seq studies [11–13, 19, 50]. Yet, a major issue in transcriptional profiling of dissociated cells, including scRNA-seq, is the evoked transcriptional perturbations that may occur during tissue dissociation, and which may bias the true detection of disease-induced transcriptional changes [20, 46]. In LCM approach, the tissue is preserved throughout the procedure and cells are isolated from dehydrated slices which prevents any procedure driven transcriptional perturbations. Accordingly, we showed very low expression of immediate early genes in isolated microglia. Although our technical approaches slightly differ, our results agree with those of Merienne et al., [54] who showed that combining LCM with RNA-seq analyses in reporter mice represents a useful approach to decrypt cell-type specific gene expression patterns. Our results further expand the value of the approach as here we show that a short staining protocol can be added to the procedure to allow isolation and transcriptome analysis of spatially distinct cell subtypes [55].

AD reactive microglia and DAM/ARM have been associated with reduced expression of microglial homeostatic genes and loss of homeostatic functions [5, 6, 11, 13]. In contrast, our results show that, across age/disease progression, gene expression of homeostatic microglial genes stays relatively stable in CM, PCM but also in PAM. Indeed, only slight down-regulation was observed in older PAM. Although these results contrast with previous analyses performed on sorted microglia [5, 6, 11, 13], they are consistent with qPCR analysis of whole cortical tissue, which shows slight down-regulation of the homeostatic genes *Pr2y12* and *Tgfb* only in 12-mo samples (reanalysis of data in [26] and data not shown). Our results also agree with a recent study that revealed that down-regulation of homeostatic microglia genes only occurs in mouse models with advanced neurodegeneration [56]. Aggregated, these results support that impairment of homeostatic functions occurs specifically in advanced stages PAM. They also support that brain dissociation procedures used in most studies may alter transcriptomic data and that homeostatic microglia functions are to a large extent preserved long after the histopathological disease hallmarks have appeared.

### Aβ plaques drive prominent alterations in PAM transcriptome

Being located close to one of the earliest and most prominent feature of AD pathology, PAM have been the subject many studies. Yet, they have mainly been investigated using either low throughput techniques (i.e., mainly imaging based, for review [57]) or indirect (i.e., top-down) medium/large throughput approaches. By combining direct isolation of PAM through LCM and RNA-seq analysis of their transcriptome, our approach provides a comprehensive understanding of molecular remodeling in this microglia subpopulation identified according to spatial criteria, without molecular or functional a priori. First, we showed that microglial proximity to amyloid plaques is the most important factor in microglia transcriptomic remodeling in this AD mouse model. These results are consistent with previous studies showing  i) enrichment, within LCM-isolated plaques tissues samples, of DEG identified in aging TgCRND8 AD mice cortical brain homogenates [22], and ii) strong transcriptomic alterations in Aβ-phagocytosing microglia [19]. Our work is also in line with a recent spatial-transcriptomic study that revealed gradual co-expression of PIG genes as a function of Aβ accumulation [21].

Extending previous findings [6, 17, 58], we showed that PAM DEGs are enriched for functions/GO-terms linked to inflammation and immune-related pathways. Moreover, we showed that PAM DEGs are enriched for the microglial reactome signature [37]. In addition to their roles in neuroinflammation processes, PAM have been associated with more neuroprotective functions in relation with Aβ processing. Indeed, they have been proposed to play key roles in Aβ encapsulation and in plaque compaction [57–60]. Consistent with PAM clustering around plaques to form a physical barrier, we identified DEGs associated with *i) chemotaxis* and *cell migration* and *ii) *cell morphology (for example *actin polymerization, actin organization, lamellipodium assembly*). These later changes are also consistent with morphological changes commonly observed in PAM. We also identified enrichment for GO-terms linked to APP processing, phagocytosis, lysosome organization, which are consistent with an involvement of PAM in Aβ clearance [5, 19]. In addition to the above functions that were mainly associated with up-regulated genes, we also identified a module of co-repressed genes which relates, at the cellular level, to synapse establishment and functioning, and at the behavioral level with learning and memory. These microglia expressed genes may represent interesting targets to restore neuronal functioning. Together with our finding that the microglial sensome signature [36] is also strongly altered in PAM, these results suggest that PAM fail to provide the appropriate support for correct synapse and neuronal functioning. These data are consistent with results showing lower response of PAM to damage signals [61] and altered spine dynamics at proximity of Aβ deposits [29].

PAM have been associated and often confounded with subpopulations of microglia identified by scRNA-seq (DAM, ARM [11, 13]) or by differential clustering (MGND [5]). Our spatial analysis of PAM firmly establishes that PAM are strongly enriched for genes of the ARM, DAM and MGnD signatures. Consistent with their close proximity to Aβ plaques, we also found a strong enrichment of PAM DEGs for the PIG signature [21]. Our data also showed a strong enrichment for the IRM signature among the PAM DEGs suggesting that IRM, for which spatial location was not previously assessed [13], are also present at proximity of the Aβ plaques. From a translational point of view, PAM are also significantly enriched for AD1/Aβ-pathology and, to a lesser extent, AD2/Tau-pathology, microglia signatures identified in postmortem human AD brains [14]. The remarkable phenotypic heterogeneity of microglia in spatially restricted plaque area was further evidenced by smFISH and immunostaining data showing that genes enriched in PAM are expressed at varying levels in different PAM of the same plaque. The origins of this local diversity remain to be established and may include paracrine regulation, cell memory mechanisms potentially involving epigenetic marks, and/or contact duration with the plaques. Although careful examination of the published data revealed that, for example, not all PAM are MHCII + [17] or that not all PAM exhibit decreased P2rY12 expression (5), to our knowledge, such local diversity around Aβ-plaques has not yet been described.

Our study also provides information relative to the temporal changes in the microglial transcriptomic remodeling in PAM, focusing on early and intermediate stage of the disease. We identified three subsets of genes that are deregulated in PAM in an age-dependent manner. In particular, we identified a small subset of genes (Brown module [28 genes]) that are up-regulated in PAM independently of age but decreased in aged CM. These genes are thus independent of the microglia aging processes and represent a unique signature of Aβ-plaques associated microglia. They are associated with the *Syndecans* reactome pathway and the *Hif KEGG signaling* pathway. Syndecans are known to bind to extracellular matrix molecules [62], and may represent a protective mechanism aimed at maintaining Aβ plaque compaction throughout the disease progression. HIF is associated with reactive oxygen species (ROS) formation, which suggests that aged PAM retain their ability to regulate ROS production. Interestingly, Hif1a signature has been recently associated with the Aβ-phagocytosing microglia subtype [19].

We also identified two other subsets (Turquoise [96 genes] and Blue [31 genes] modules), which contain genes that are, respectively, globally up-regulated and down-regulated in PAM and which showed same direction of changes in older control microglia. Such matching deregulations in older CM and PAM are consistent with aging and AD sharing similar molecular mechanisms, with AD representing an accelerated aging. Our finding that these two gene subsets exhibited less deregulation in older compared to younger PAM would be consistent with cell exhaustion or “burn out”. Interestingly, this concept is consistent with results from PET imaging studies in the APP^SL70^ AD mouse model showing that Aβ-PET binding increased as a function of age, whereas TSPO-PET binding had an inverse U-shape growth function, thus indicating that microglial activity decreases relative to ongoing amyloidosis [63]. Functional studies are needed to investigate the hypothesis of cell exhaustion in older PAM.

### PCM, morphologically intact but nevertheless reactive

In contrast to PAM, PCM have been less well studied in AD studies. When studied, this subpopulation was generally compared to PAM rather than CM. Here, the use of LCM provided us with the unique opportunity to comprehensively characterize PCM in AD, and to compare their transcriptome to both CM and PAM.

We defined PCM as high GFP+ expressing cells located more than 100 µm away from any TR+ plaque. These criteria are similar to those used by Rothman et al. [22] to identify brain tissues associated with plaques. Because thiazine-red stains dense-core Aβ plaques, we cannot exclude the possibility that at least some of the microdissected PCM were associated to more diffuse Aβ deposits that are TR- but would have been 6E10+ (see Additional file 12: Fig. S12B, C). To minimize this possibility, we paid attention to target isolated microglia during microdissection, thus excluding microglia showing any sort of clustering (Additional file 12: Fig. S12C). Factors driving PCM reaction may be multiple, ranging from contact with soluble or oligomeric Aβ species to sensing pathology-driven changes in neuronal activities. Exploring these factors will require further studies. One important observation is that although PCM overall morphology is not changed compared to control microglia (present data and [15]), this microglia subtype displayed a time-dependent increase in gene deregulation that correlates with disease progression. This dynamic deregulation mirrors the global increase of the neuroinflammatory status observed in the cortex of both mice and humans as the disease progress [26, 64], and parallels the increase in soluble Aβ fractions in this AD mouse line (data not shown). By showing specific deregulations in PCM, we demonstrated that this microglia subtype is not a by-stander but rather plays significant roles in AD progression.

Overall our results suggest that, in addition to densely aggregated Aβ species, microglia may also react to other factors including Aβ small oligomers or possibly monomers. Although other cellular communications (i.e., altered neuron–microglia crosstalk) could be involved in PCM deregulations, microglial detection and reaction to small oligomers are highly relevant to AD progression as these oligomers are more bioactive at synapses and drive stronger microglia reaction (65). Our results are also consistent with data correlating tissue transcriptomic changes to 6E10 staining and showing a gradual increase in the network connectivity of PIG [21]. Enrichment of PIG in the PCM signature supports that the expression of the so-called “plaque-induced genes” are in fact not restricted to dense Aβ plaques. Similarly, our results show that genes deregulated in PCM are also enriched for the DAM/ARM/MGnD/IRM/AD1 and AD2 mouse and human microglia signatures. This was further confirmed by our in situ results demonstrating in PCM expression of *Cst7* and *Clec7a*, two ARMs markers, and of *Cybb*, including CLEC7A in human samples. Thus, our data revealed that although DAM/ARM/MGnD and IRM are further represented around Aβ plaques, these microglia subtypes are likely to be involved in disease progression on a more general scale. Our data provide a new and comprehensive overview of the potential role of PCM in AD progression and identified several hub/key genes. Indeed, functional and gene network analysis of genes deregulated in PCM revealed that these cells are reactive and involved in the immune response. Identification of *H2aa*, *Cd68* and *Ctsd* acting as hub genes in PCM suggest that this microglia subtype is involved in antigen presentation and proteins processing pathways [66]. Interestingly, overexpression of these genes from 4-mo APP^swe^/PS1dE9 mice in PCM indicates that these processes are activated from the very early stage of the disease. Microglial *Axl* and *Merkt* TAM receptor tyrosine kinases have recently been shown to be essential mediators of Aβ plaques recognition and engulfment and it was proposed that TAM-driven phagocytosis promotes rather than inhibits dense-core plaque development [59]. Up-regulation of *Axl* in PCM from early/intermediate stages suggests that it may also be involved in plaque formation, thus representing a neuroprotective mechanism. We also identify Ephrin-B3 (*Efnb3*) as being up-regulated specifically in PCM compared to both CM and PAM. Ephrin-B3 is a transmembrane ligand of Ephrin receptors that mediate a myriad of essential cellular processes, including immune cell activation [67]. Interestingly, increased microglial expression of *Efnb3* was recently demonstrated in experimental autoimmune encephalomyelitis (EAE). Specifically, *Efnb3* was shown to be involved in microglia–astrocytes interactions in the context of inflammation in EAE [68]. Further studies are warranted to explore whether Ephrin-B3 is involved in the recruitment of microglia to plaque forming area, activation of microglia and/or astrocytes. More broadly, this supports that PCM are involved in early Aβ plaque formation and opens new area of research to decipher the functional roles of this microglia subtype.

Our transcriptomic data also revealed that PCM display an inflammatory profile that build-up as the pathology progresses. Importantly, we showed that PCM reactive phenotype starts from the early stages when Aβ load is small and plaque density low (i.e., less than 1 plaque/mm^2^ re-analysis from [26]). Consistent with these results, Sierksma et al. [69] revealed up-regulation of inflammation related genes from 4-mo in the APP^swe^/PS1^L166P^ AD mouse hippocampus and Sobue et al. [56] showed moderate early microglial dysfunction in AD precuneus in patients. These molecular findings also correlate with in vivo positron emission tomography (PET) studies suggesting biphasic neuroinflammation response in patients, with the earliest peak occurring at a prodromal stage of the disease [64, 70]. However, it remains to be determined whether the changes in the inflammation status observed at this early stage are associated with a global up-regulation of inflammatory genes in PCM or whether it corresponds to the occurrence of neuropathogenic niches.

## Conclusions

We demonstrate that LCM combined with RNA-seq allows us to analyze transcriptome remodeling in spatially distinct cells without preconceived notions of the molecular and/or functional changes that would affect these cells. Our unique and unbiased strategy thus usefully complements previous results obtained using tissue destructive approaches. Our data confirm and extend previous studies on the role of PAM in AD progression; they offer a comprehensive and temporal view of the molecular changes in these cells, focusing on early and intermediate stages of the disease in non-aggressive AD mouse model (i.e., as compared to the 5XFAD model that was most often used). As a whole, our results support that PAM may have both positive and negative impacts on the surrounding tissues. They may represent a physical barrier to prevent small Aβ oligomers spreading, but are also releasing pro-inflammatory cytokines that are deleterious for the surrounding cells and are less supportive for synaptic functions. Importantly, our study reveals for the first time that, although PCM display a homeostatic-like phenotype, those microglia subtype are reactive, engaged in specific biological processes and are likely to participate to the disease progression. Our data suggest that these microglia sub-population may be key for detecting small Aβ oligomers and initiating plaque formation. Although PCM are less enriched than PAM for AD-associated genes, they may represent interesting cellular targets. Indeed, one can speculate that if their protective roles (i.e., plaque formation) can be promoted while their pro-inflammatory functions are prevented, then disease progression could be impeded. Finally, we reveal further molecular heterogeneity in both PAM and PCM. Further work is needed to understand how this very local heterogeneity builds-up, to identify its dynamics, and to determine the consequences for the disease progression, particularly in AD-vulnerable regions such as neocortex and hippocampus. Deciphering these mechanisms will allow to target specific subpopulation of microglia with the ultimate goal of promoting beneficial microglial functions and alleviating deleterious ones.

### Supplementary Information

**Additional file 1: Figure S1.** Study design and validation of the approach **(A)** Detailed scheme of the protocol. Brain slice preparation: After lethal Euthasol® injection, mouse brain was rapidly perfused 20 ml PBS followed by 20 ml PBS containing 20% sucrose. The brain was then removed, immersed overnight in PBS solution containing 30% sucrose and then flash frozen in − 40 °C Isopentane. Frozen cryopreserved brains were cut in 8 µm coronal slices in clean cryostat and mounted on glass slides. Slice staining and dehydration: On the day of experiment, slices were rapidly stained with Thiazin Red and then progressively dehydrated. Laser microdissection: Cells of interest were (1) identified based on GFP expression and distance from TR+ plaques under a fluorescent laser-capture microdissection microscope, and (2) laser captured using an infrared laser. RNA extraction: totRNA from pooled captured microglia from each animal/condition was extracted. **(B)** Linear regression plot between normalized expression values detected in FACs isolated microglia (*x*-axis, in log_2_; Hirbec et al., 2018) and normalized RNA-seq pseudocounts in LCM control microglia (*y*-axis, in log_2_; present study) for 103 genes, showing high correlation between the two approaches. **(C)** Heatmap of Pearson correlations between each sample, showing stronger correlation coefficients between samples of the same group than from intergroup correlations.

**Additional file 2: Figure S2. **Gene expression of specific microglial genes in the different microglia subpopulations. (A) Homeostatic genes; (B) DAM genes; (C) ARMs genes; (D) IRM genes. Control microglia (CM, greens), APP/PS1 plaque-distant microglia (PCM, oranges) and plaque-associated microglia (PAM, purples). Shades code for age: 4-mo, light color; 8-mo, median color; and 12mo, dark color. Statistics shown on the graph are from RNAseq analyses of PAM *vs* CM (Table-S5), PCM *vs* CM (Table-S7) and PAM *vs* PCM (Table-S8), respectively.

**Additional file 3: Figure** **S3.** Schematic representation of the bioinformatic analyses workflow.

**Additional file 4: Figure S4. **Microglial gene expression remodeling in plaques-associated microglia** (A, E)** Boxplots of eigengene values for each sample in PAM versus CM deregulated genes. **(A)** Turquoise module genes (1639 genes). Statistical analyses: 2-ways ANOVA with *Microglia subtype* and *Age* as between subjects’ factors. *Subtype* *p* < 0.001; *Age* *p* < 0.05. FDR corrected post hoc tests; ***p < 0.001, **p < 0.01. **(C-D)** Cytoscape network visualization of highly connected genes in the three largest subnetworks of turquoise module. 1st **(B)**, 2nd **(C)** and 3rd **(D)** largest highly subnetworks **(E)** Blue module genes (212 genes). Statistical analyses: 2-ways ANOVA with *Microglia subtype* and *Age* as between subjects’ factors. *Subtype* *p* < 0.001. FDR corrected post hoc tests; **p < 0.01. **(F)** Cytoscape network visualization of highly connected genes in the blue module. In gene netwoks, log_2_ expression ratio between PAM and CM are mapped to the nodes using a blue–white–red gradient with red indicating higher expression in PAM, and blue lower expression. The inner and outer rings represent log-ratio in, respectively, the 8- and 12-mo samples. Yellow nodes depict hub genes within the network.

**Additional file 5: Figure S5. **PAM and CM transcriptome remodeling are affected by age.** (A)** Venn diagram showing the overlap between DEGs in PAM versus CM (FDR < 0.05, purple circle) and genes showing differential expression in *Microglia-subtype:Age* interaction (GLM analyses, raw *p*-value < 0.05, blue circle). **(B)** Boxplots of eigengene values for each sample in the three WGCNA gene modules identified within the 179 age-dependent PAM versus CM DEGs. Statistical analyses: 2-ways ANOVA with *Microglia subtype* and *Age* as between subjects’ factors; Turquoise module (96 genes), *Subtype* *p* < 0.001; *Microglia subtype*:*Age* *p* < 0.001; Blue module (31 genes), *Subtype* *p* < 0.001; *Microglia subtype*:*Age* *p* < 0.01; Brown module (28 genes), *Microglia-Subtype* *p* < 0.001; Age *p* < 0.01, *Microglia subtype*:*Age* *p* < 0.001. FDR corrected post hoc tests; *** *p* < 0.001, ** p < 0.01. **(C)** GO and pathways analyses (*p* < 0.05; Term size filter [10–500]) for the Turquoise, Blue and Brown gene modules. BP: GO-biological process; K: KEGG pathways; R: Reactome Pathways.

**Additional file 6: Figure S6. **Microglial gene expression remodeling in plaque-distant AD microglia (PCM). **(A)** Confocal images of CM in CX3CR1^+/eGFP^ (left) or APP/PS1^Tg/0^: CX3CR1^+/eGFP^ (right) cortices. Grey squares represent zoomed region with 3D reconstruction, showing that global morphology is not affected at this stage. Scale bar 50 µm. **(B)** Principal component analysis (PCA) of gene expression in the different microglia samples, based on the 13,711 expressed genes **(C)** Venn diagram showing the overlap between DEGs in PCM versus CM (*p*_raw_ < 0.05, orange circle) and genes showing differential expression in *Microglia-subtype:Age* interaction (GLM analyses, *p*_raw_ < 0.05, blue circle). **(D)** Boxplots of eigengene values for each sample in the two WGCNA gene modules identified within the 595 age-dependent PCM versus CM DEGs. Statistical analyses: 2-ways ANOVA with *Microglia subtype* and *Age* as between subjects’ factors; Turquoise module (213 genes): *Subtype* *p* < 0.001; *Microglia subtype*:*Age* *p* < 0.001; Blue module (146 genes): Age *p* < 0.05; *Microglia subtype*:*Age* *p* < 0.01. FDR corrected post hoc tests; ***p < 0.001, **p < 0.01, *p < 0.05. **(E)** GO and pathways analyses (*p* < 0.05; Term size filter [10–500]) for the Turquoise and Blue gene modules. BP: GO-biological process; K: KEGG pathways; R: Reactome Pathways.

**Additional file 7: Figure S7. **PAM vs PCM, age-dependent transcriptomic changes.** (A)** Venn diagram showing the overlap between DEGs in PAM versus PCM (FDR < 0.05, green circle) and genes showing differential expression in *Microglia-subtype:Age* interaction in PAM and PCM samples (GLM analyses, *p*_raw_ < 0.05, blue circle). WGCNA analysis identified a module of 91 co-deregulated genes (turquoise). **(B)** Cytoscape representation of deregulated GO terms (“Biological Process” category) in age-dependent PAM *vs* PCM deregulated genes (Turquoise module, 91 genes). **(C)** Boxplots of eigengene values for each sample in age-dependent PAM *vs* PCM deregulated genes (Turquoise module, 91 genes). Statistical analyses: 2-ways ANOVA with *Microglia subtype* and *Age* as between subjects’ factors. *Subtype* *p* < 0.001; *Subtype:Age* *p* < 0.001. FDR corrected post hoc tests; ***p < 0.001, **p < 0.01. **(D)** Cytoscape network visualization of highly connected genes in age-dependent PAM versus PCM DEGs (Turquoise module, 91 genes). Log_2_ expression ratio between PAM and PCM are mapped to the nodes using a blue–white–red gradient with red indicating higher expression in PAM, and blue lower expression. The inner and outer rings represent log-ratio in, respectively, the 8- and 12-mo samples. **(E)** Efnb3 expression in the different microglia subpopulations Control microglia (CM, greens), APP/PS1 plaque-distant microglia (PCM, oranges) and plaque-associated microglia (PAM, purples). Shades code for age: 4-mo, light color; 8-mo, median color; and 12mo, dark color. Statistics shown on the graph are from RNA-seq analyses of PAM *vs* CM (Table-S5), PCM *vs* CM (Table-S7) and PAM *vs* PCM (Table-S8), respectively.

**Additional file 8: Figure S8. **Gene deregulations in CM, PCM and PAM for the different gene signatures identified. (A) Heatmap of PAM *vs* CM DEGs: relative expression in CM, PCM and PAM along aging (related to Fig. 2); (B) Heatmap of PCM *vs* CM DEGs: relative expression in CM, PCM and PAM along aging (related to Fig. 3); (C) Heatmap of PAM *vs* PCM DEGs: relative expression in CM, PCM and PAM along aging (related to Fig. 4).

**Additional file 9: Figure S9.** Clec7a/Dectin-1 protein expression in cortical microglia of 4-mo APP^swe^/PS1^dE9^ mice. Microglia detection (IBA1, green), Dectin-1 staining (red) and 6E10/Aβ deposits (white) were combined on slices from 4-mo WT or APP^swe^/PS1^dE9^ mice. We observed increased Dectin-1 staining in PAM, but also in some PCM. As with RNAscope, we noted heterogeneity in Dectin-1 expression in both PAM and PCM. Scale bar 20 µm.

**Additional file 10: Figure S10. **Cst7 mRNA expression in control (CM); plaque-distant (PCM) and plaque-associated (PAM) microglia. Microglia detection (GFP, green), Thiazine Red (TR) staining for amyloid plaques (red) and *Cst7* mRNA expression (purple) were combined on brain slices in **(A)** APP/PS1^Tg/0^: CX3CR1^+/eGFP^ or **(E)** CX3CR1^+/eGFP^ mouse. **(A)** 12-mo APP/PS1^Tg/0^:CX3CR1^+/eGFP^ mouse cortical field exhibiting multiple amyloid plaques. Plaque-associated microglia (PAM) are located within 70 µm (purple dotted circle) of the plaque epicenter; while plaque-distant microglia (PCM) are located farther than 100 µm (orange dotted circle) from the plaque epicenter. *Cst7* mRNA expression is absent in control microglia **(E)** but can be evidenced in both plaque-associated **(A, C)** and plaque-distant **(A, D)** microglia. White arrowheads point to *Cst7* positive plaque-distant microglia. **(B)** Pseudocounts of *Cst7* expression in CM (greens), PCM (oranges) and PAM (violets) along ages. **(C)** Zoomed region around TR+ plaque showing *Cst7* overexpression in PAM. Note the heterogeneity for *Clec7a* expression among PAM, blue arrowhead for no expressing microglia, yellow arrowhead for low expressing microglia, grey arrowhead for high expressing microglia. **(D)** Zoomed region of *Cst7* positive plaque-distant microglia. Scale bar 20 µm.

**Additional file 11: Figure S11. **Cybb mRNA expression in control (CM); plaque-distant (PCM) and plaque-associated (PAM) microglia. Microglia detection (GFP, green), Thiazine Red (TR) staining for amyloid plaques (red) and *Cybb* mRNA expression (purple) were combined on brain slices in **(A)** APP/PS1^Tg/0^: CX3CR1^+/eGFP^ or **(E)** CX3CR1^+/eGFP^ mouse. **(A)** 12-mo APP/PS1^Tg/0^:CX3CR1^+/eGFP^ mouse cortical field exhibiting several amyloid plaques. Plaque-associated microglia (PAM) are located within 70 µm (purple dotted circle) of the plaque epicenter; while plaque-distant microglia (PCM) are located farther than 100 µm (orange dotted circle) from the plaque epicenter. *Cybb* mRNA expression is absent in control microglia **(E)** but can be evidenced in both plaque-associated **(A, C)** and plaque-distant **(A, D)** microglia. White arrowheads point to *Cybb* positive plaque-distant microglia. **(B)** Pseudocounts of *Cybb* expression in CM (greens), PCM (oranges) and PAM (violets) along ages. **(C)** Zoomed region around TR+ plaque showing *Cybb* overexpression in PAM. Note the heterogeneity for *Cybb* expression among PAM, blue arrowhead for no expressing microglia, yellow arrowhead for low expressing microglia, grey arrowhead for high expressing microglia. **(D)** Zoomed region of *Cybb* positive plaque-distant microglia. Scale bar 20 µm.

**Additional file 12: Figure S12. **Thiazin-red versus 6E10 staining to detect amyloid deposits in brain tissues samples. 4-mo APP/PS1::CX3CR1^+/egfp^ were co-stained with Thiazin-Red (TR, red channel) that best labels dense Aβ plaques, 6E10 that can recognize both dense and non-dense forms of Aβ (6E10, purple channel) and GFP (green channel) to identify microglia. (**A**) All TR^+^ Aβ deposits were also 6E10^+^. TR^+^ / 6E10^+^ represented about 90% of all Aβ deposits and were surrounded by several microglia. (**B-C**) About one-tenth of the 6E10^+^ deposits were TR^−^. (**B**) In most of the cases, these 6E10^+^/TR^−^ deposits were associated with clustered microglia, which therefore did not meet our selection criteria for PCM. (**C**) On some occasion 6E10^+^/TR^−^ deposits were associated with isolated microglia. Overall, isolated microglia in the vicinity of 6E10^+^/TR^−^ are rare, representing less that 3% of all Aβ deposits in 4-mo APP^swe^/PS1^dE9^::CX3CR1^+/egfp^,, however these cells did meet our selection criteria for PCM. Scale bar 30 µm.

**Additional file 13: Table S1**. Summary of the preliminary tests performed to establish the protocol for tissue preparation. Optimized conditions used in the present suty are highlighted in green.

**Additional file 14: Table S2. **List of the samples included in the study.

**Additional file 15: Table S3. **Expression values for all expressed genes (in pseudocounts), related to Fig. 2.

**Additional file 16: Table S4. **Fold-change of typical microglial homeostatic genes in XO4+; DAM; ARM; MGnD and PAMs (NS: non significant; −: not reported).

**Additional file 17: Table S5. **Differentially expressed genes in PAM vs CM, related to Fig. 2. Ta ble S5B: GO analysis of the PAM vs CM DEGs Turquoise module, related to Fig. 2C. Table S5C: GO analysis of the PAM vs CM DEGs Blue module, related to Fig. 2D.

**Additional file 18: Table S6. **PAM vs CM, Genes networks and subnetworks (highlighted in green: hub genes).

**Additional file 19: Table S7. A. **Differentially expressed genes in PCM vs CM, related to Fig. 3. Table S7B: GO analysis of the PCM vs CM DEGs, related to Fig. 3C.

**Additional file 20:** **Table S8.** A: Differentially expressed genes in PAM vs PCM, related to Fig. 4A. Table S8B: GO analysis of the PAM vs PCM DEGs Turquoise module, related to Fig. 4C.

**Additional file 21: Table S9.** Differentially expressed genes in PCM vs CM excluding DEGs in PAM vs CM, related to Fig. 4D. Table S9B: Differentially expressed genes in PAM vs PCM excluding DEGs in PAM vs CM, related to Fig. 4D.

**Additional file 22: Table S10. **GO analysis of the PAM vs CM:Age DEGs Turquoise module, related to Figure S7B, C.

**Additional file 23: Table S11.** Human postmortem samples used in the study (Highlighted in grey, samples processed for RNAscope)

## Data Availability

All data generated or analyzed during this study are either included in this published article or in its supplementary information files. Datasets generated during the current study are available in the Gene Expression Omnibus (GEO) repository, GSE205048.

## Extended methods

### Laser capture microdissection: cells selection and microisolation

1. After staining and dehydration, preparation strips (Arcturus) were applied onto the glass slides containing the cryosections to remove destroyed tissues and/or non-specifically stuck materials;
2. Glass slides containing the sections were placed on the microscope stage over the illuminated area;
3. Images of the zone of interest were first acquired under red-fluorescence microscopy; virtual circles of 70 µm (PAMs selection) or 100 µm (PCMs selection) radius, centered on each TR^+^ plaques, were drawn on the visualizing monitor using the Arcturus software.
4. Fluorescence illumination was then switched to the green channel to visualize GFP+ cells (i.e., microglial cells), keeping on screen the circles drawn under the red fluorescence channel;
5. A CapSure HS LCM cap was automatically placed on the area of interest;
6. Cells of interest were identified as such: PAM were defined as GFP+ cells located within the 70 µm radius disc, whereas PCM were GFP+ cells located beyond any circle of 100 µm radius. Further, for PCMs we excluded cells that, even in absence of TR+ staining, appeared clustered;
7. Once identified on the screen, the cell of interest was positioned under the IR-laser, which was then fired. The same operation was repeated until all desired cells within the useful surface of the Capsure cap have been captured. Of note, the laser characteristics were set at the smallest “spot” size (i.e., 7.5 µm); the power of the Infra-red laser, the number and the durations of the pulses were adjusted for each slide;
8. After cells of interest within the zone were captured, the cap was lifted, and captured cells were observed on the QC station. When unwanted tissue was removed together with a cell of interest, the Cap was replaced on a clean glass slide and the unwanted tissue was fired using the Arcturus UV-laser for destruction.
9. When the cap was cleared from any unwanted material, it was inserted onto a 0.5-ml microcentrifuge tube containing RLT-plus buffer (Qiagen; #1053393) and immediately stored at − 80 °C.

### Primers used in qPCR experiments

**Table:**

Genes	Forward primer	Backward primer
CLEC7a	CCAGGATAGCTGTTGTTTCAGAG	CCAAGCATAGGATTCCCAAA
CYBB	CACAGGCCTGAAACAAAAGA	GCTTCAGGTCCACAGAGGAA
CST7	TCTGCTGCCTGGTCTTGAG	TCACACGTGAGTTAAGGTCCTG

## Abbreviations

AD: Alzheimer's disease
Aβ: Amyloid-β
PAM: Plaque-associated microglia
PCM: Plaque distant microglia
CM: Control microglia
GWAS: Genome-wide association studies
DAM: Disease-associated microglia
LRM: Late response microglia
ARM: Activated response microglia
ERM: Early response microglia
IRM: Interferon-response microglia
TRM: Transiting response microglia
MGnD: Microglial neurodegenerative phenotype
AD1: Amyloid-β pathology-associated human microglia
AD2: Tau pathology-associated human microglia
IAM: Inflammatory-associated microglia
PIG: Plaque-induced gene
MHCII+: Class II major histocompatibility complex
FACS: Fluorescent-activated cell sorting
PFA: Paraformaldehyde
TR: Thiazine red
RINs: RNA integrity numbers
RLE: Relative log expression
DEG: Differentially expressed genes
GLM: Generalized linear models
PCA: Principal component analysis
WGCNA: Weighted gene co-expression network analysis
EPC: Edge percolated component
MCC: EcCentricity Maximal Clique Centrality
MNC: Maximum neighborhood component
GO: Gene Ontology
KEGG: Kyoto Encyclopedia Gene and Genomes
LCM: Laser capture microdissection
FDR: False discovery rate
GLM: Generalized Linear Model
TNF: Tumor necrosis factor
smFISH: Single-molecule fluorescence in situ hybridization
ROS: Reactive oxygen species
scRNA-seq: Single-cell RNA sequencing
snRNA-seq: Single-nuclei RNA sequencing
NA: Not analyzed
NCI: Non-cognitively impaired
